# Eutectogels as a Semisolid Electrolyte for Organic Electrochemical Transistors

**DOI:** 10.1021/acs.chemmater.3c02385

**Published:** 2024-02-05

**Authors:** Yizhou Zhong, Naroa Lopez-Larrea, Marta Alvarez-Tirado, Nerea Casado, Anil Koklu, Adam Marks, Maximilian Moser, Iain McCulloch, David Mecerreyes, Sahika Inal

**Affiliations:** † Organic Bioelectronics Laboratory, Biological and Environmental Science and Engineering Division, King Abdullah University of Science and Technology (KAUST), Thuwal 23955-6900, Saudi Arabia; ‡ POLYMAT, University of the Basque Country UPV/EHU, Avenida Tolosa 72, Donostia-San Sebastian, Guipuzcoa 20018, Spain; § IKERBASQUE, Basque Foundation for Science, Plaza Euskadi 5, Bilbao 48009, Spain; ∥ Department of Chemistry, University of Oxford, Oxford OX1 3TF, U.K.

## Abstract

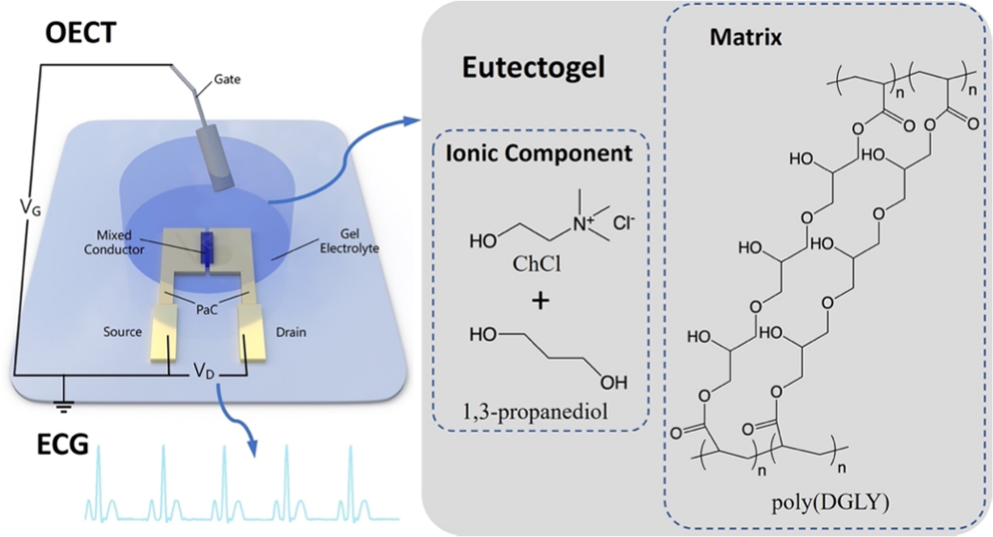

Organic electrochemical transistors (OECTs) are signal transducers offering high amplification, which makes them particularly advantageous for detecting weak biological signals. While OECTs typically operate with aqueous electrolytes, those employing solid-like gels as the dielectric layer can be excellent candidates for constructing wearable electrophysiology probes. Despite their potential, the impact of the gel electrolyte type and composition on the operation of the OECT and the associated device design considerations for optimal performance with a chosen electrolyte have remained ambiguous. In this work, we investigate the influence of three types of gel electrolytes—hydrogels, eutectogels, and iongels, each with varying compositions on the performance of OECTs. Our findings highlight the superiority of the eutectogel electrolyte, which comprises poly(glycerol 1,3-diglycerolate diacrylate) as the polymer matrix and choline chloride in combination with 1,3-propanediol deep eutectic solvent as the ionic component. This eutectogel electrolyte outperforms hydrogel and iongel counterparts of equivalent dimensions, yielding the most favorable transient and steady-state performance for both p-type depletion and p-type/n-type enhancement mode transistors gated with silver/silver chloride (Ag/AgCl). Furthermore, the eutectogel-integrated enhancement mode OECTs exhibit exceptional operational stability, reflected in the absence of signal-to-noise ratio (SNR) variation in the simulated electrocardiogram (ECG) recordings conducted continuously over a period of 5 h, as well as daily measurements spanning 30 days. Eutectogel-based OECTs also exhibit higher ECG signal amplitudes and SNR than their counterparts, utilizing the commercially available hydrogel, which is the most common electrolyte for cutaneous electrodes. These findings underscore the potential of eutectogels as a semisolid electrolyte for OECTs, particularly in applications demanding robust and prolonged physiological signal monitoring.

## 1 Introduction

The organic electrochemical transistor (OECT) has been a popular biosignal transducer to build wearable electronics due to the soft mechanical properties of (semi)conducting polymers used in the channel, high local amplification of the transistor circuit, and the design flexibility and processability of its components.^1^ The OECT has three main components: the gate electrode, the semiconducting channel, and the electrolyte, each affecting the signal-to-noise ratio (SNR), the resolution, and the stability of signals recorded by the OECT.^2−4^ While much work has been done to correlate the device performance metrics to the channel material properties, less attention has been paid to the electrolyte, which is often an aqueous salt. For wearable applications of the OECT, solid-state or gel-like electrolytes are more convenient, limiting electrolyte leakage, volatilization, and corrosion of electronic parts.^5^ These solid-like electrolytes should have high ionic conductivity (although most suffer from low conductivities leading to slow OECT switching speeds) and be biocompatible, conformable, and stable.^6−11^

Hydrogels and iongels are two common gel-based electrolytes used in OECTs, which incorporate aqueous electrolytes or ionic liquids (ILs) within an organic matrix, respectively.^12,13^ The soft nature of hydrogels and the possibility to tune their physicochemical properties (such as mechanical strength, elasticity, flexibility, and stimuli responsiveness) make them interesting to build OECTs with different functionalities.^14^ The hydrogels integrated with OECTs are typically based on cross-linked polymer networks such as poly(ethylene oxide) (PEO), poly(hydroxyethyl acrylate) (PHEA), poly(hydroxyethyl methacrylate) (PHEMA), and poly(vinyl alcohol) (PVA) or biopolymers (e.g., type A and B gelatins).^15−19^ Swelling the hydrogel network in saline solutions, such as sodium chloride (NaCl) or phosphate-buffered saline (PBS), renders the materials ionically conducting. However, the ion-bearing hydrogel structure is easily distorted at high temperatures and upon long-term use, with a change in ionic conductivity due to water evaporation.^20,21^ As an alternative to hydrogels, IL gels (iongels) have shown promise as a solid matrix bearing nonvolatile ions with melting point below 100 °C.^7,22−26^ The common ILs used in these gels include 1-ethyl-3-methylimidazolium ethyl-sulfate ([C2MIM][EtSO4]), 1-butyl-3-methylimidazolium bis(trifluoromethylsulfonyl)imide ([BMIM][TFSI]), and 1-ethyl-3-methylimidazolium bis(trifluoromethylsulfonyl)imide ([EMIM][TFSI]), but their toxicity and high fluorine content preclude their use in direct contact with skin and the environment.^7,23,24^ Biocompatible ILs have been developed, comprising cholinium cations together with amino acids or carboxylic acids as anions (e.g., cholinium lactate ([Ch][Lac]) or cholinium glycolate ([Ch][Glyco])).^27,28^ These iongels were embedded inside PVA-based gels through -H bonding or photopolymerized using the IL monomer, showing the highest ionic conductivities from 1 × 10^–3^ to 1 × 10^–2^ S/cm, biocompatibility, and superior ambient stability. All of these features make these materials highly attractive for long-term cutaneous electrophysiology and other biomedical applications.^29^ Cholinium-based iongels were also used as electrolytes to implement all-solid-state OECTs but have not operated with channels other than poly(3,4-ethylenedioxythiophene):poly(styrenesulfonate) (PEDOT:PSS).^25,26^

Deep eutectic solvents (DESs) are a new generation of environmentally green and inexpensive ionic compounds—analogs of ILs.^30,31^ DESs are systems formed from a combination of hydrogen bond acceptors (HBAs) and hydrogen bond donors (HBDs), which can contain a variety of anionic and/or cationic species.^32^ Usually, HBAs include quaternary ammonium chloride and metal salts (choline chloride (ChCl), betaine, ZnCl_2_, AlCl_3_, etc.) and HBDs include polyols, polyacids, and polyamines (ethylene glycol, glycerol, urea, etc.).^33^ Among these, the combination of ChCl with polyhydric alcohols or polyacids such as lactic acid, glycolic acid, glycerol, and 1,3-propanediol is the class that has been most studied due to their high melting point and thus liquid state at room temperature.^34^ These ChCl-based DESs show low vapor pressure, high conductivity, stability, biodegradability, and nonflammability^20^ and can be blended with other materials such as PEDOT:PSS to achieve printable electrodes.^35^ They are also used to form a gel-like network, similar to iongels, namely, eutectogels.^34,36^ Despite these interesting properties, to the best of our knowledge, eutectogels have never been integrated with OECTs.

In this work, we developed these three classes of gel electrolytes, with similar polymeric matrices and three different ionic components (salty water, IL, and DES), and evaluated their performance as the OECT dielectric medium to uncover the origins of electrolyte-type-dependent device characteristics. We found that the eutectogel electrolyte made of poly(glycerol 1,3-diglycerolate diacrylate), poly(DGLY), as the polymer matrix, and ChCl and 1,3-propanediol DES as the ionic component outperformed the hydrogel and iongel samples when integrated into p-type depletion, p-type, and n-type enhancement mode OECTs. The eutectogel-gated enhancement mode OECTs exhibited excellent stability in long-term (simulated) electrocardiogram (ECG) signal monitoring, with no degradation in signal-to-noise ratio (SNR) upon 5 h of continuous operation as well as during 30 days of daily measurements. Compared to devices relying on a commercially available hydrogel electrolyte, the eutectogel-gated OECTs demonstrated higher signal amplitude and SNR, highlighting the potential of eutectogels as a nonliquid electrolyte for applications requiring a long-term and robust ion reservoir.

## 2 Results and Discussion

**Figure 1:**
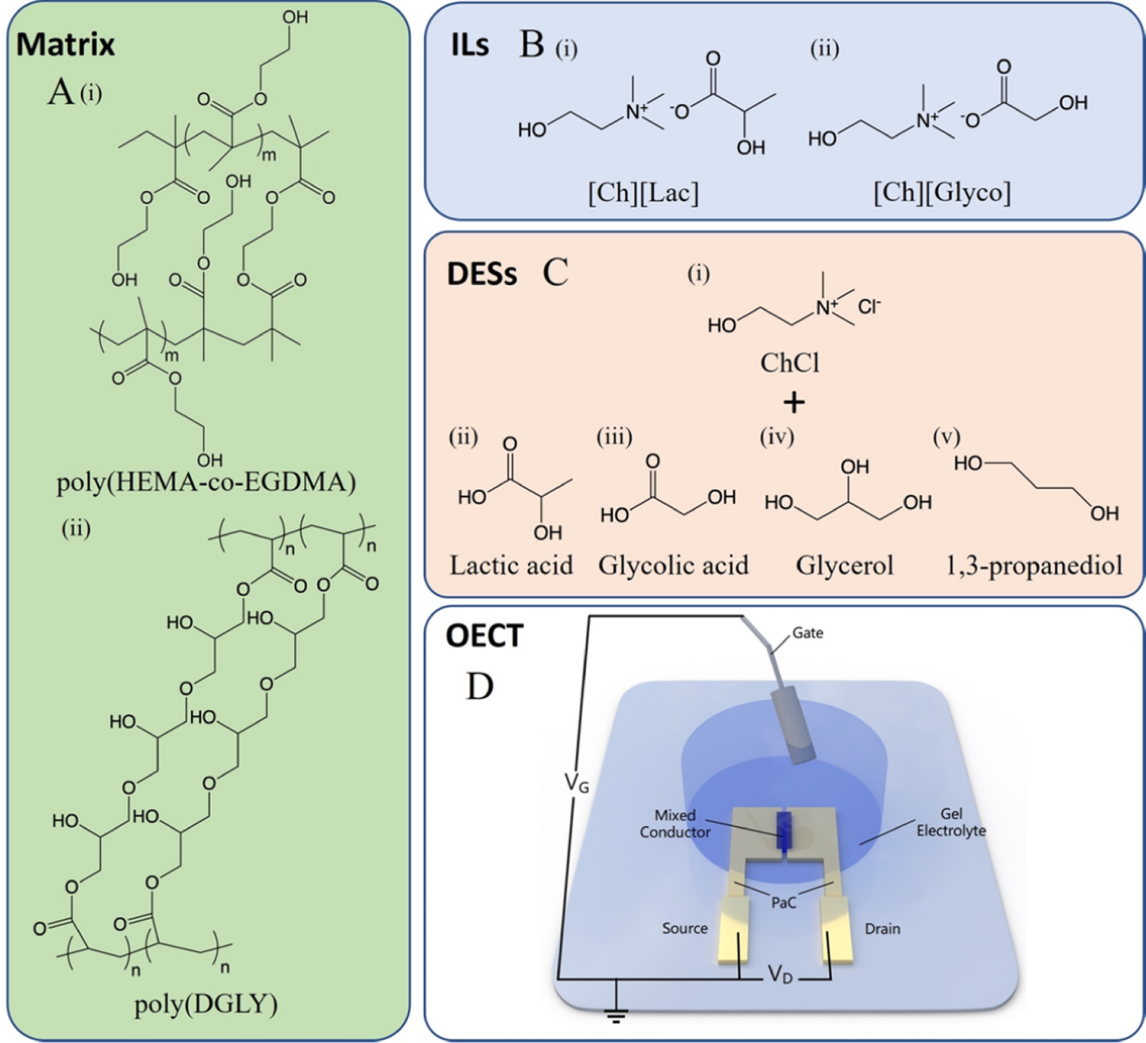
Chemical structures of the gel electrolyte components and the OECT architecture. The chemical structures of (A) the polymeric matrices: (i) poly(HEMA-*co*-EGDMA) and (ii) poly(DGLY), (B) the ionic components used in the iongel samples: (i) [Ch][Lac] and (ii) [Ch][Glyco], and (C) the ionic components eutectic used in eutectogel samples: (i) ChCl as the HBA, and (ii) lactic acid, (iii) glycolic acid, (iv) glycerol, and (iv) 1,3-propanediol as the HBDs. (D) The OECT configuration.

In this work, we developed seven gel electrolyte samples, including one hydrogel, two iongels, and four eutectogels, and evaluated their performance as nonliquid electrolytes for OECTs. Table 1 summarizes the composition of all our nonaqueous electrolytes. To make the hydrogel, we photopolymerized 2-hydroxyethyl methacrylate (HEMA) and the cross-linker ethylene glycol dimethacrylate (EGDMA) and obtained the polymer matrix (poly(HEMA-*co*-EGDMA), Figure 1A-i). We swelled the polymer in 0.5 M PBS to generate the ionic hydrogel, namely, HG, which has a polymer matrix–ionic component ratio of 50–50 wt %. We used PBS to introduce ions inside the gel so that a fair performance comparison between HG and PBS can be made.

**Table 1:** Gel Samples and Their Components with the Corresponding Ratios

sample	gel type	polymer matrix	ionic component	composition
HG	hydrogel	poly(HEMA-*co*-EGDMA)	PBS	50 wt % polymer, 50 wt % PBS
IL1	iongel	poly(DGLY)	[Ch][Lac]	30 wt % polymer, 70 wt % IL
IL2			[Ch][Glyco]	
DES1	eutectogel	poly(DGLY)	ChCl:lactic acid^a^	30 wt % polymer, 70 wt % DES
DES2			ChCl:glycolic acid^a^	
DES3			ChCl:glycerol^a^	
DES4			ChCl:1,3-propanediol^a^	

a The mol ratio of HBA (ChCl) to HBD (lactic acid, glycolic acid, glycerol, and 1,3-propanediol) is 1:2.

The iongels and the eutectogels bear the same polymer matrix, poly(glycerol 1,3-diglycerolate diacrylate) (poly(DGLY), Figure 1A-ii). To make iongel samples IL1 and IL2, we integrated [Ch][Lac] (Figure 1B-i) and [Ch][Glyco] (Figure 1B-ii), respectively, into poly(DGLY). The 70 wt % of the iongels are made of biocompatible cholinium cations and amino acids or carboxylic acids used as anions, with a perspective to build devices with the gel interfacing the skin.^37,38^ To obtain eutectogel samples with various HBDs, we used biocompatible ChCl-based DESs (Figure 1C-i), namely, DES1 (ChCl:lactic acid, Figure 1C-ii), DES2 (ChCl:glycolic acid, Figure 1C-iii), DES3 (ChCl:glycerol, Figure 1C-iv), and DES4 (ChCl:1,3-propanediol, Figure 1C-v). The eutectogels DES1 and DES2 comprise the same ions as in iongels IL1 and IL2, respectively (Ch^+^ and Lac^–^ for DES1 and IL1, Ch^+^ and Glyco^–^ for DES2 and IL2). We included DES3 and DES4 in the series since these can make more neutral eutectogels than DES1 and DES2.^33,39^ Similar to iongels, all eutectogels contain 70 wt % of ionic components in their composition. Finally, HG, IL1, and IL2 have neutral pH, while the components of DES1 and DES2 exhibit strong acidity, and those of DES3 and DES4 are weak acids (Figure S1).

Figure 1D displays the architecture of the OECT, which contains a micrometer-scale channel with a length of 10 μm and a width of 100 μm. We used three (semi)conducting polymers as the channel material to evaluate the best channel material/electrolyte couple, including the p-type conducting polymer PEDOT:PSS, the p-type semiconducting polymer p(g_3_C_2_T2-T), and the n-type semiconducting polymer p(C_6_NDI-T) (see chemical structures in Figure S2). We selected these materials as they all lead to high transconductance (*g*_m_) OECTs when operated in aqueous electrolytes.^2,40^ We characterized the performance of the OECT with each of the three channels gated through the gel samples using silver/silver chloride (Ag/AgCl) as a nonpolarizable gate electrode. All of the electrolytes had the same geometry, i.e., they were cut as cylinders with a diameter of 8 mm and a height of 0.5 mm (Figure S3).

### 2.1 OECT Performance

#### 2.1.1 Hydrogel-Gated OECTs

**Figure 2:**
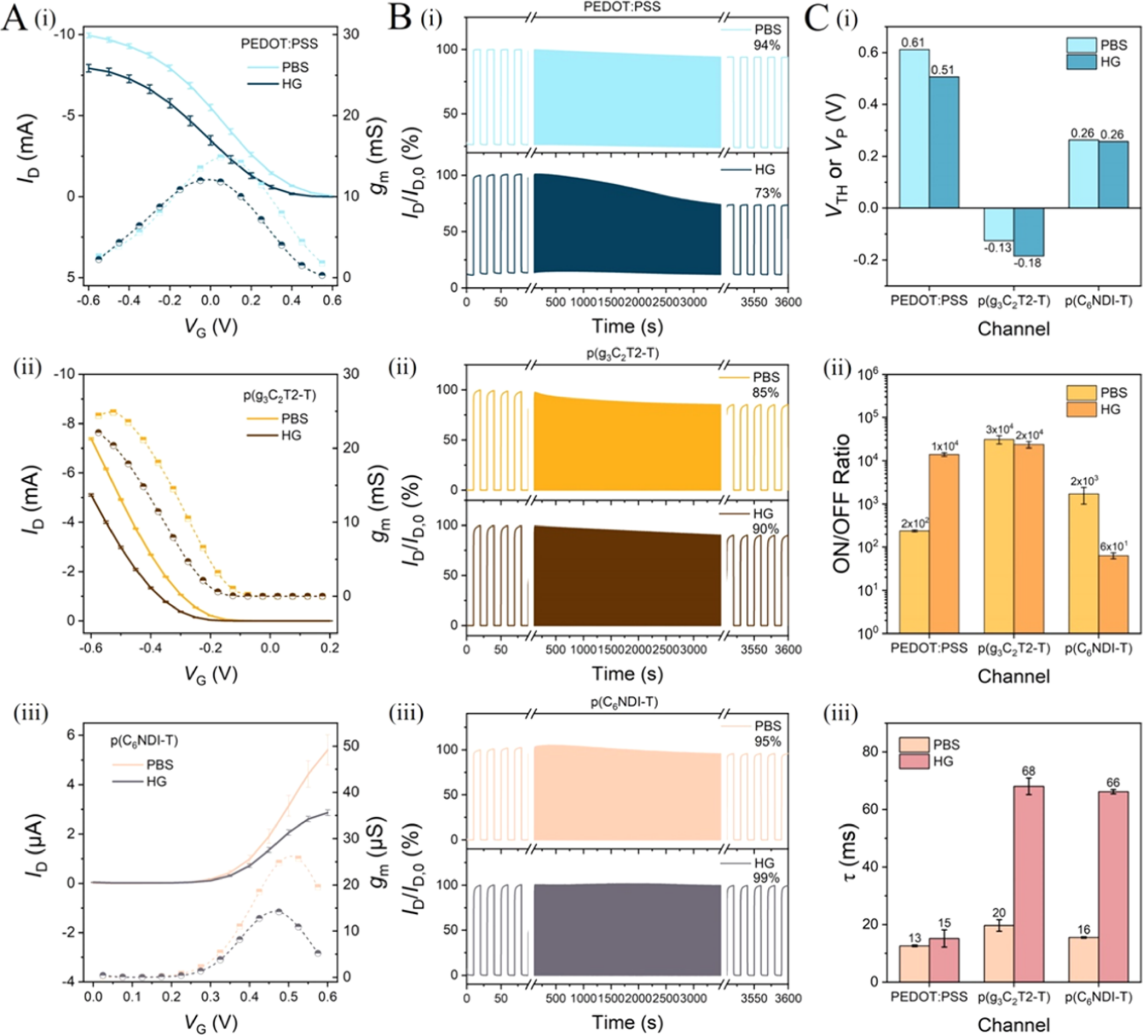
Comparison of the hydrogel-gated OECT characteristics with those of PBS-gated devices. (A) Transfer curves (solid lines), *g*_m_ characteristics (dashed lines), and (B) 1 h operational stability of OECTs with channels made of (i) PEDOT:PSS, (ii) p(g_3_C_2_T2-T), and (iii) p(C_6_NDI-T). We evaluated the operational stability performance of the OECTs by switching the channel OFF and ON with 10 s spent at each state, i.e., ca. 180 seconds-long doping and dedoping cycles. We calculated the *I*_D_ retention (%) at the end of these measurements. (C) (i) Threshold voltage (*V*_TH_) for enhancement mode OECTs and pinch-off voltage (*V*_P_) for the PEDOT:PSS OECT, (ii) ON/OFF ratios, and (iii) response time (τ) of all devices. All characteristics were recorded at *V*_D_ = −0.5 V for p-type OECTs and *V*_D_ = 0.5 V for n-type OECTs.

**Figure 3:**
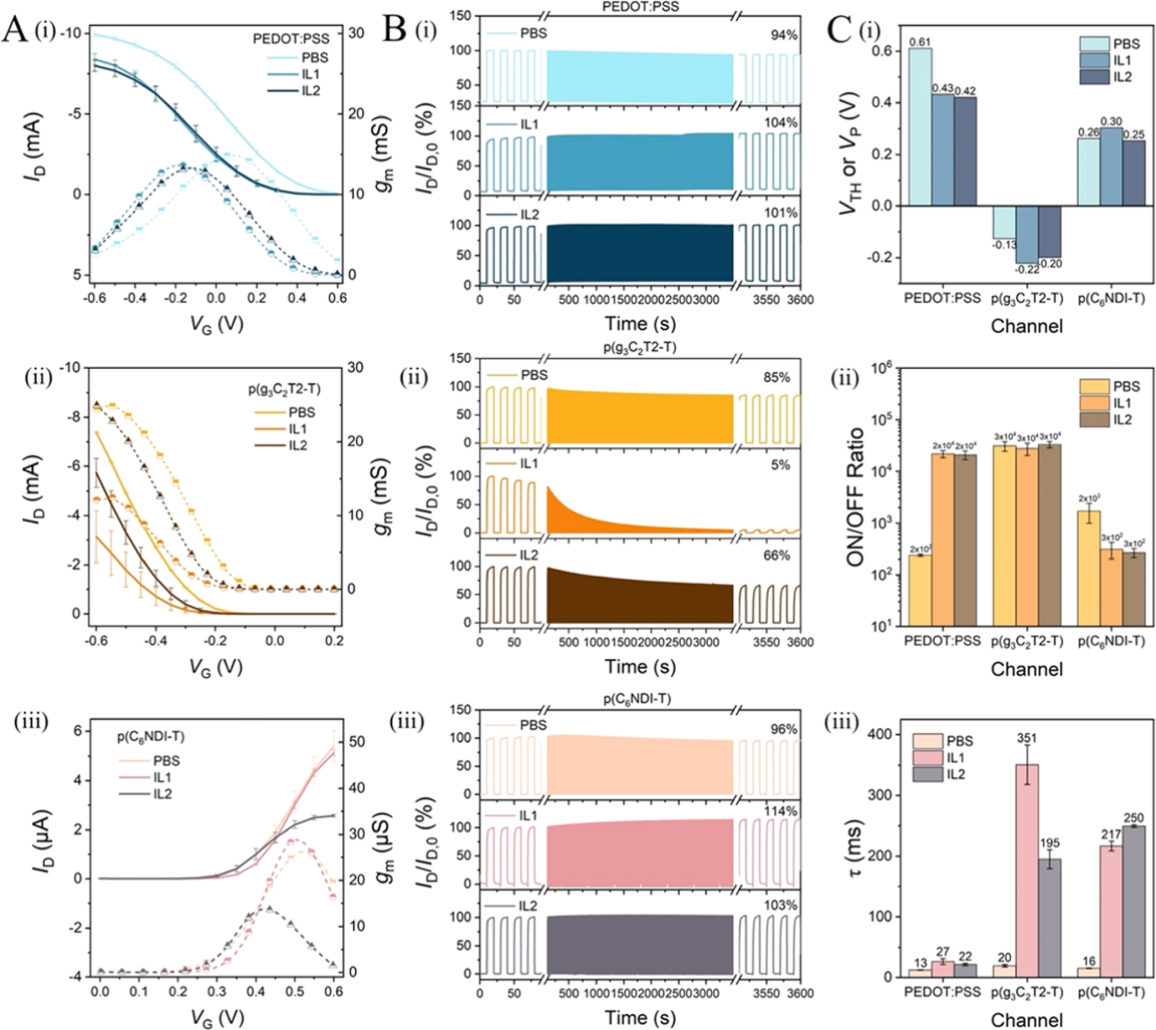
Comparison of the iongel-gated OECT characteristics with those of PBS-gated devices. (A) Transfer curves (solid lines), *g*_m_ characteristics (dashed lines), and (B) 1 h operational stability of OECTs with channels made of (i) PEDOT:PSS, (ii) p(g_3_C_2_T2-T), and (iii) p(C_6_NDI-T) films. (C) (i) *V*_TH_ and *V*_P_, (ii) ON/OFF ratios, and (iii) τ values of all devices. All characteristics were recorded at *V*_D_ = −0.5 V for p-type OECTs and *V*_D_ = 0.5 V for n-type devices.

**Figure 4:**
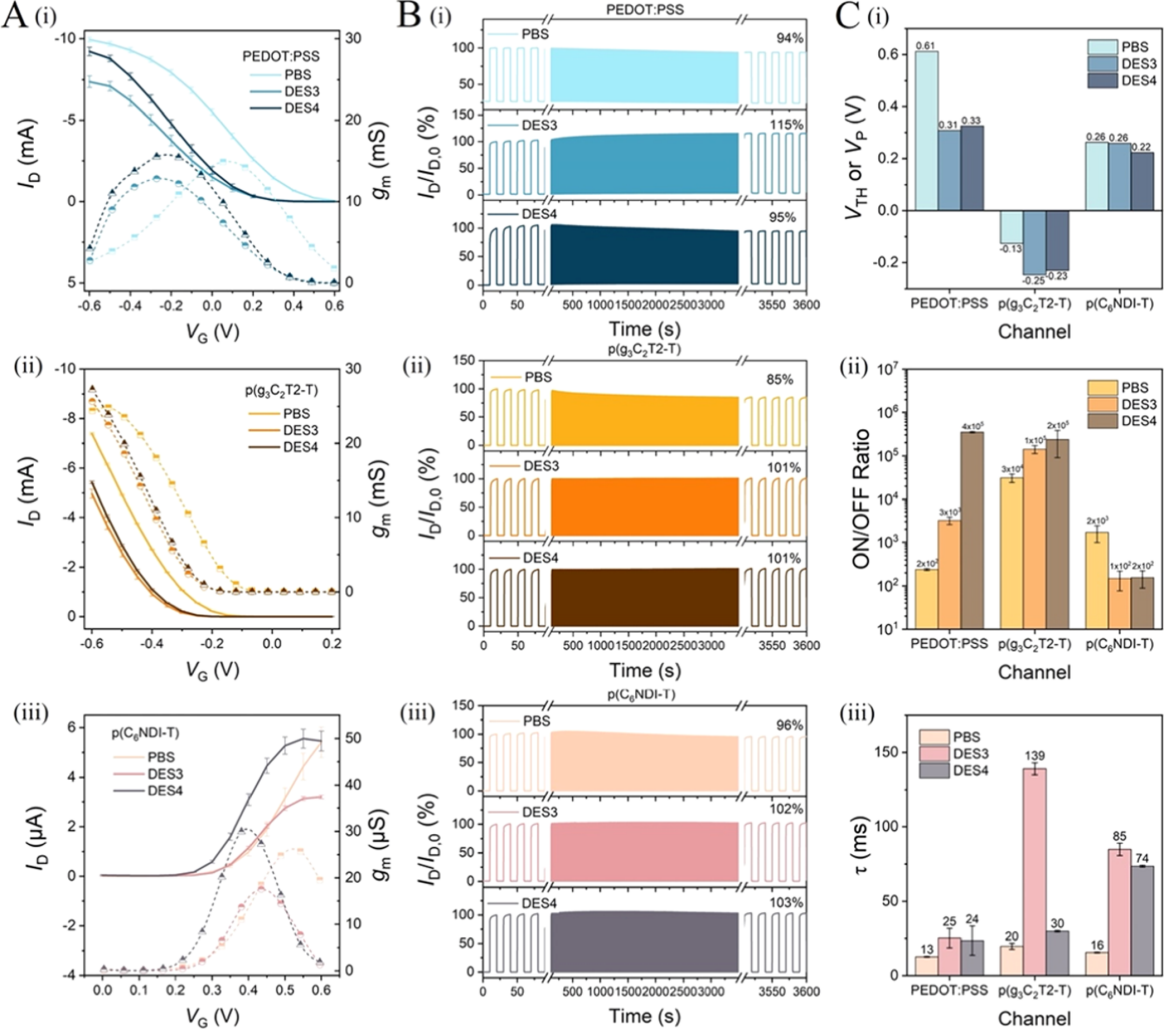
Comparison of eutectogel-gated (DES3 and DES4) OECT characteristics with those of PBS-gated devices. (A) Transfer curves (solid lines), *g*_m_ characteristics (dashed lines), and (B) 1 h operational stability: (i) PEDOT:PSS, (ii) p(g_3_C_2_T2-T), and (iii) p(C_6_NDI-T) OECTs. (C) (i) *V*_TH_ and *V*_P_, (ii) ON/OFF ratios, and (iii) τ values for all three channels gated with eutectogels or PBS. All characteristics were recorded at *V*_D_ = −0.5 V for p-type OECTs and *V*_D_ = 0.5 V for n-type devices.

The output characteristics (channel current versus drain voltage (*I*_D_–*V*_D_) curves) of hydrogel-gated OECTs seem similar to what we obtain with PBS alone (Figure S4), yet with quantitative differences. For example, in both electrolytes, the PEDOT:PSS OECT is inherently ON at the zero bias gate voltage (*V*_G_) and requires a high positive *V*_G_ (e.g., 0.6 V) to switch completely OFF (Figure S4A). The enhancement mode devices are “OFF” at *V*_G_ = 0 V, and they need a *V*_G_ to push anions or cations into the polymeric channel to compensate for the electronic charges injected from the contact and, consequently, turn the device ON (Figure S4B,C). The ON current of the n-type OECT is 3 orders of magnitude smaller than that of p-type devices (∼μA versus ∼mA), independent of the electrolyte, due to the lower electronic conductivity of the n-type semiconductor.^41^Figure 2A depicts the corresponding transfer curves and *g*_m_ characteristics, the former showing how the OECT toggles between ON and OFF states with respect to *V*_G_ and the latter informing about the voltage range the device reaches its maximum amplification (*g*_m_ = ∂*I*_D_/∂*V*_G_). When we compare the performance of hydrogel-gated devices with that of PBS-gated ones, some differences appear: (1) the maximum *I*_D_ and *g*_m_ of hydrogel-gated OECTs are lower than those with PBS for all three types of OECT channels (Figure 2A), (2) the PEDOT:PSS OECT has a significant *I*_D_ drop at the end of the operational stability measurement with the hydrogel, while the other two channels are stable (Figure 2B), (3) hydrogel-gated OECTs have a more negative threshold (*V*_TH_) or pinch-off voltage (*V*_P_) compared to PBS-gated devices for two p-type channels (0.51 versus 0.61 V for PEDOT:PSS, −0.18 versus −0.13 V for p(g_3_C_2_T2-T), while the *V*_TH_ of the n-type device is independent of the electrolyte (0.26 V), Figure 2C-i), (4) hydrogel-gated OECTs have a higher ON/OFF ratio when the channel is PEDOT:PSS, while they have a lower ON/OFF ratio if it is p(C_6_NDI-T) (Figure 2C-ii), and (5) hydrogel-gated OECTs are slower than PBS-gated OECTs (Figure 2C-iii), evident from the higher response time (τ) values extracted from the current–time profiles (Figure S5).

#### 2.1.2 Iongel-Gated OECTs

Both iongel samples (IL1 and IL2) gate all of the OECT channels (Figure S6). This result is exciting as n-type OECTs are often unsuitable for gating with commercially available ILs and their gels. For example, we show in Figure S7A that p(C_6_NDI-T) OECTs cannot be gated by either the IL [EMIM][TFSI] (the most common IL for p-type OECTs)^9,23,42^ or its gel cross-linked by poly(vinylidene fluoride-*co*-hexafluoropropene) (PVDF-HFP) (see the chemical structures in Figure S7B). Figure 3 demonstrates the performance comparison of iongel-gated OECTs with PBS-gated devices. We find that (1) the maximum *I*_D_ and *g*_m_ measured with iongels are lower than those with PBS (Figure 3A), except for the p(C_6_NDI-T) OECT working with IL1, which has comparable *I*_D_ and *g*_m_ values with the PBS-gated channel, (2) the p(g_3_C_2_T2-T) OECTs have significant drop in *I*_D_ at the end of the operational stability measurement with both iongel samples, while the other two materials are stable with the iongels (Figure 3B), (3) iongel-gated OECTs have more negative *V*_TH_ (or *V*_P_) compared to PBS-gated devices for two p-type channels, while the *V*_TH_ of n-type devices is independent of the electrolyte (Figure 3C-i)—which was also the case for the hydrogel gating, (4) iongel-gating increases the ON/OFF ratio of the PEDOT:PSS OECTs while decreasing the ON/OFF ratio of the p(C_6_NDI-T) device (Figure 3C-ii)—a similar trend with the hydrogel gating, (5) iongel-gated OECTs switch ON and OFF slower than the PBS-gated devices (Figure 3C-iii; see Figure S8 for the current–time profiles)—the same trend we observed with hydrogel-gated devices.

We next evaluated whether the channels have performance differences depending on the ion type inside the gels. We highlight that all ON/OFF ratios and *V*_TH_ are independent of the IL type. For PEDOT:PSS devices, none of the characteristics are affected by the gel type. For the n-type film, IL1 gating results in moderately higher currents and *g*_m_ than IL2 gating, with all other performance metrics being the same. The most striking difference between the gels is observed during the biasing stress measurements of the p(g_3_C_2_T2-T) devices. Although IL1-gated channels almost completely degrade with an *I*_D_ retention of 5%, devices with IL2 maintain 66% of the initial channel current after 1 h of continuous biasing (Figure 3B-ii). Note also that the IL2-gated p(g_3_C_2_T2-T) OECTs have higher *I*_D_ and *g*_m_ levels than the IL1-gated devices (Figure 3A-ii) and they switch ON much faster than IL1-gated devices (Figure 3C-iii). IL2, therefore, presents a better gel material for all types of OECTs. We hypothesize that the poor long-range stability of p-type enhancement mode OECTs gated by the iongels originates from the large size of their anions (Lac^–^ for IL1 and Glyco^–^ for IL2), which could perturb the morphology of the p(g_3_C_2_T2-T) film by getting trapped inside.

#### 2.1.3 Eutectogel-Gated OECTs

Evaluating the OECT output characteristics with the four eutectogel electrolytes shows that p(C_6_NDI-T) channels gated with DES1 and DES2 have extremely low current outputs (Figure S9). DES3 and DES4, on the other hand, lead to outstanding device characteristics for all three channel materials. The maximum *I*_D_ measured with eutectogel-gated devices is lower than that with PBS-gated ones (a conclusion valid for all nonliquid electrolytes), except for the DES4-gated p(C_6_NDI-T) OECT achieving higher *I*_D_ than the PBS-gated analog (Figure 4A). Moreover, the maximum *g*_m_ achieved by some gel-channel combinations is higher than that gated in PBS: DES4 results in higher gains for all three channel materials, and DES3 combined with p(g_3_C_2_T2-T) outperforms the others (Figure 4A). Both DES3- and DES4-gated OECTs show stable currents with all three types of channels (Figure 4B). These devices have more negative *V*_TH_ (or *V*_P_) compared to PBS-gated devices for two p-type channels, while the *V*_TH_ of n-type devices turn ON around the same voltages (Figure 4C-i). The ON/OFF ratio of the eutectogel-gated p-type channels is higher than PBS-gated counterparts, while for the n-type channel, we observe a decrease in the ON/OFF ratio (Figure 4C-ii). As for the switching speeds, just like other nonliquid electrolytes, eutectogel gating leads to slower devices (Figures 4C-iii and S10). DES4 leads to better-performing devices compared to DES3 with higher *I*_D_ and *g*_m_ (Figure 4A), higher ON/OFF ratios (Figure 4C-ii), and faster switching speeds (Figure 4C-iii) for all three channel materials.

### 2.2 Searching for the Best-Performing Gel Electrolyte

**Figure 5:**
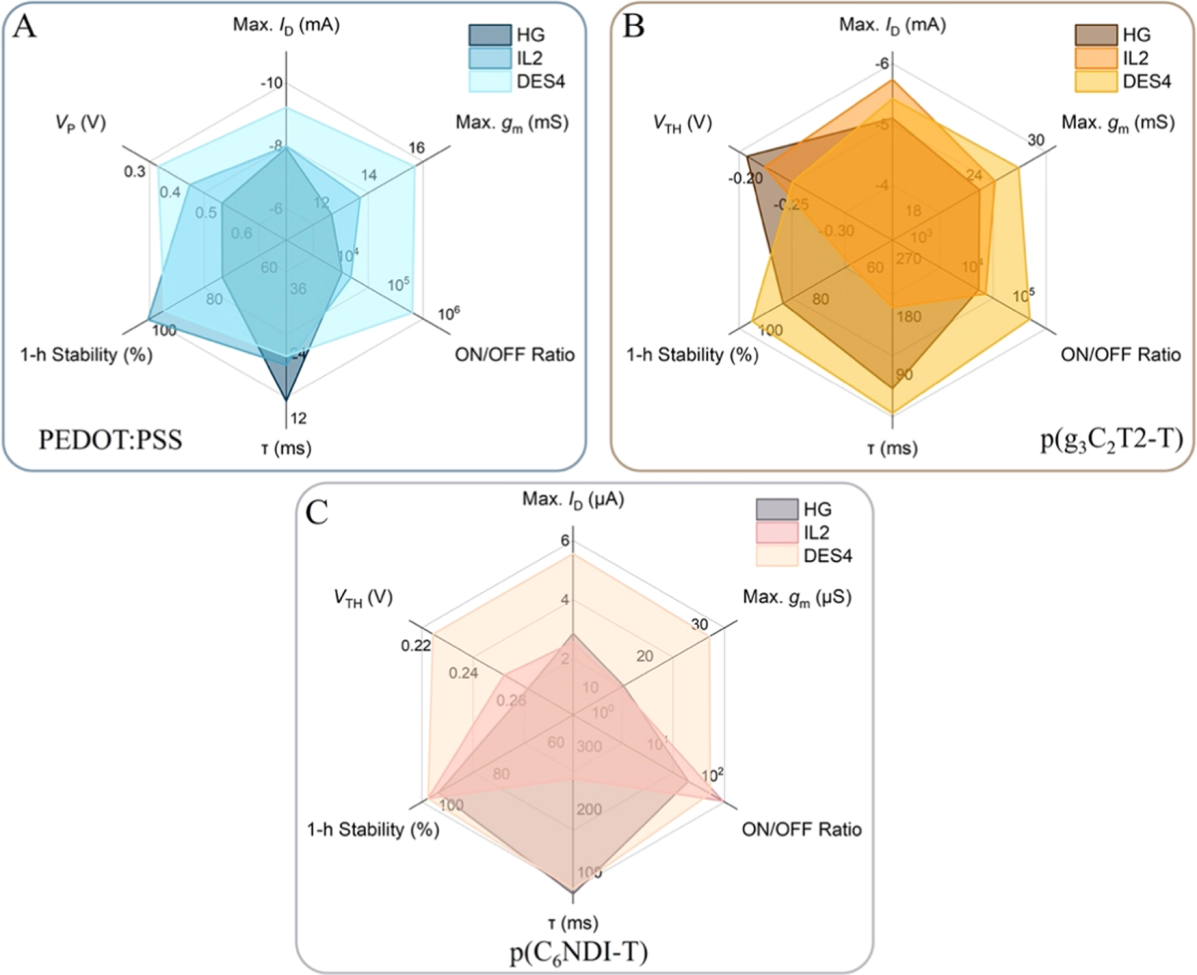
OECT performance comparison based on the type of gel electrolyte (HG, IL2, and DES4) when the channel is (A) PEDOT:PSS, (B) p(g_3_C_2_T2-T), or (C) p(C_6_NDI-T).

Table S1 summarizes the OECT characteristics, i.e., the maximum *I*_D_ reached saturation, the maximum *g*_m_, ON/OFF ratio, switching speed, the current stability after 1 h of continuous operation, and the *V*_TH_ extracted for each gel class that led to maximum performance for all channels (HG, IL2, and DES4). In Figure 5, we show these performance metrics by using radar plots. For PEDOT:PSS channels, the eutectogel gating leads to the best-performing OECTs (Figure 5A). The hydrogel-gated device is the fastest, while the differences among devices are only marginal (the fastest device speed is 15.2 ms, and the slowest one with DES4 is 23.5 ms). For the p(g_3_C_2_T2-T) channel, the results are similar: the eutectogel gating leads to devices with the highest *g*_m_, the ON/OFF ratio, the 1 h stability, and the fastest switching speed (Figure 5B). Hydrogel gating allows for the earliest *V*_TH_, but the DES4 gating is only marginally different (*V*_TH_ with the hydrogel is −0.18 V, and the one with DES4 is −0.23 V). The eutectogel gating also leads to the best n-type device performance (Figure 5C; see Figure S11 for a performance comparison between PBS- and DES4-gated devices).

Based on these results, we conclude that eutectogel-gating (DES4), independent of the chosen channel material, leads to the best-performing devices compared to iongel and hydrogel gating. Note that the gels in our work always led to slower devices compared to those gated with PBS. We postulate that the slow speed can be attributed not only to the cross-linked network of the gels, which obstructs the ion drift, but also to the low ionic conductivity of the gels. Figure S12 shows conductivity levels of 10^–5^ S/cm for iongels, 10^–4^ S/cm for hydrogels, and 10^–3^ S/cm for eutectogels, all lower than that of PBS (10^–2^ S/cm). The low ion migration rate in solid electrolytes compared to that of liquid ones typically leads to a slower switching speed in OECTs.^9,43−45^ One possible approach to improving the switching speed is the preloading of the gel ions inside the channel, which allows us to bypass the ion penetration step and avoid long-range ion motion.^46−49^

### 2.3 What Makes the Eutectogel the Best-Performing Nonliquid Electrolyte?

#### 2.3.1 Gate Electrode Electrochemical Potential Depends on the Electrolyte Type

**Figure 6:**
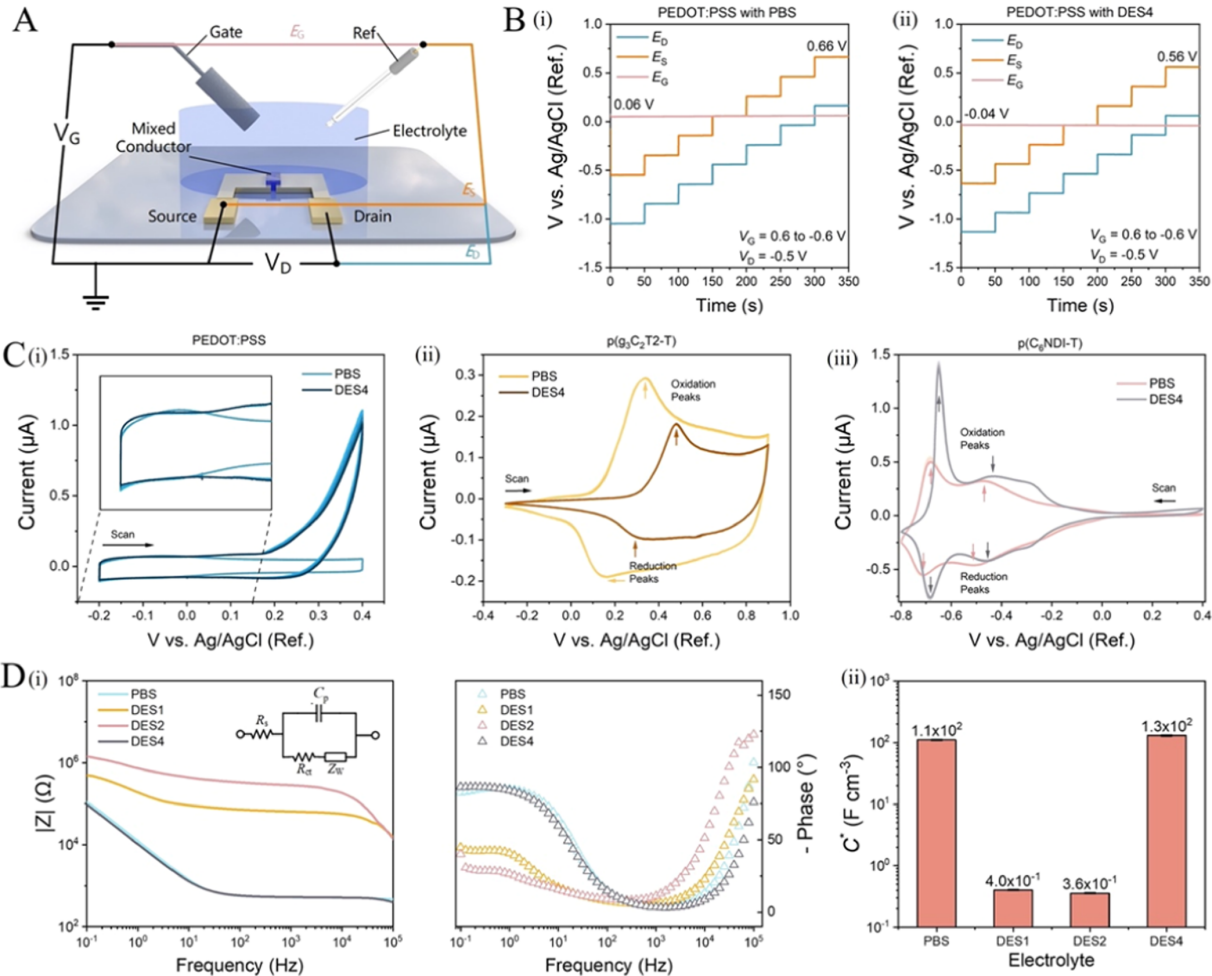
Electrochemical potentials of OECT terminals and the electrochemical characteristics of polymer films in different electrolytes. (A) The multichannel potentiostat setup to monitor in-operando electrochemical potential of device terminals with respect to the Ag/AgCl reference electrode. (B) Electrochemical potential changes at the PEDOT:PSS OECT contacts measured during operation in (i) PBS and (ii) DES4 gel. *V*_D_ was at −0.5 V and *V*_G_ changed from 0.6 to −0.6 V with a step of 0.2 V. The gate potential during the entire duration of the measurement (*E*_G_) and the source potential at *V*_G_ = −0.6 V (*E*_S_) are labeled. (C) Cyclic voltammetry (CV) curves of a (i) PEDOT:PSS, (ii) p(g_3_C_2_T2-T), and (iii) p(C_6_NDI-T) film recorded in PBS or DES4. The scan rate was 50 mV/s. (D) (i) Bode plot of a p(C_6_NDI-T) film recorded in PBS or eutectogels. The inset in panel (i) shows the equivalent circuit model used to fit the impedance spectra, consisting of the electrolyte resistance (*R*_s_), the polymer capacitance (*C*_p_), the charge-transfer resistance (*R*_ct_), and the Warburg impedance (*Z*_W_). (ii) The volumetric capacitance of the p(C_6_NDI-T) film when doped in PBS or eutectogels.

The OECT channel current is regulated by the magnitude of the effective gate voltage (*V*_G,eff_), which is affected by the nature of the electrolyte. We can estimate *V*_G,eff_ by measuring the in-operando electrochemical potential of each transistor terminal in a given electrolyte with respect to a stable Ag/AgCl reference electrode by using a multichannel potentiostat (see the schematic of the setup in Figure 6A). To operate our OECTs, we fix the *V*_D_ at |0.5 *V*| and increase the magnitude of *V*_G_ with a step of |0.2 *V*|. The OECT electrochemical potentials should follow the variations in the applied voltage at the gate electrode. The potential difference between the gate and the source (*E*_G_ – *E*_S_) corresponds to *V*_G_, and the drain-to-source potential difference (*E*_D_ – *E*_S_) corresponds to *V*_D_. Since our gate electrode, Ag/AgCl, is ideally a nonpolarizable electrode, *E*_G_ remains mostly constant during operation and is equivalent to the open circuit potential (OCP) of the electrode, which renders *V*_G,eff_ = −*E*_S_.

For PEDOT:PSS OECTs, we find that the gate has an OCP of 0.06 V in PBS (Figure 6B-i) and −0.04 V in DES4 (Figure 6B-ii). This result means that the channel will be gated at a larger *V*_G,eff_ in PBS than in DES4 at the same operating conditions (e.g., −0.66 versus −0.56 V when *V*_G_ = −0.6 V), which generates a higher channel ON current. The same measurement done for the p-type enhancement mode device reveals that in PBS, the channel can be brought to higher doping states with the same *V*_G_ applied in PBS compared to that in DES4 (Figure S13A). The effect of the gel on the *V*_G,eff_ is, however, the opposite when the channel is the n-type material. The negative OCP value of the gate electrode in DES4 causes a larger *V*_G,eff_ at the same *V*_G_ (e.g., 0.65 versus 0.55 V at *V*_G_ = 0.6 V and *V*_D_ = 0.5 V; Figure S13B), thereby resulting in higher ON currents when the gel is used as the electrolyte.

#### 2.3.2 Oxidation/Reduction Peak Positions Change with the Electrolyte Type

Figure 6C shows the cyclic voltammetry (CV) curves of the polymer thin films recorded in PBS and DES4 where the electrolyte type is observed to change the positions of the oxidation and reduction peaks. For example, we recorded an earlier oxidation onset for the p(g_3_C_2_T2-T) film in PBS compared to that in DES4 (Figure 6C-ii). The *V*_TH_ of the devices seems to follow this trend (−0.13 V in PBS versus −0.23 V in DES4; Figure 4C-i). The p(C_6_NDI-T) thin film has its reduction and oxidation peaks occurring at lower potentials in DES4 compared to that in PBS (Figure 6C-iii), a trend reflected in the *V*_TH_ values. As the *V*_TH_ shifts based on the electrolyte, so does the *V*_G_ where the maximum *g*_m_ occurs.

The CV curve of PEDOT:PSS in PBS has a rectangular shape, indicating its capacitive nature (Figure 6C-i).^50^ The CV curves of PEDOT:PSS in both electrolytes seem similar, except that in DES4, the current increases at positive voltages without an associated reduction peak, indicative of irreversible reactions between PEDOT:PSS and ions in DES4. We, however, did not observe any negative impact of these plausible reactions on the performance of the OECT, including its stability.

#### 2.3.3 Charging Ability Depends on the Gel Composition

The electrochemical potentials of OECT terminals and CV curves help us understand why some electrolyte-channel combinations allow for higher OECT performance compared to others. There is, however, also a need to understand why OECTs with some other electrolyte-channel combinations do not operate effectively. For instance, we noted that despite bearing the same polymer network and the same HBA with DES4 (and DES3), DES1 and DES2 eutectogels are unable to turn ON p(C_6_NDI-T) OECTs. To rule out the fact that these differences may be related to a transport-related phenomenon in the channel, we used electrochemical impedance spectroscopy (EIS) to estimate the anion–electron coupling capacity when the films are addressed through these electrolytes. The polymer capacitance (*C*_p_) was estimated by fitting the impedance spectra (Figure 6D-i) using the equivalent circuit model (Figure 6D-i, inset), and the C* was calculated by normalizing *C*_p_ with the volume of the film investigated. We quantified the volumetric capacitance (C*) of the p(C_6_NDI-T) film with the four electrolytes PBS, DES1, DES2, and DES4 (as a representative “high performance” DES), using EIS. Figure 6D-ii shows that while the C* of the p(C_6_NDI-T) film in DES4 has comparable values to that in PBS (∼10^2^ F/cm^3^), the C* in DES1 and DES2 is lower by 3 orders of magnitude (10^–1^ versus 10^2^ F/cm^3^). These results are in agreement with our OECT results and suggest that the cations of DES1 and DES2 fail to interact with the film.

We showed that IL1, IL2, and DES4 can effectively gate the p(C_6_NDI-T) channel, whereas DES1 and DES2 cannot. These results are surprising as (1) eutectogels DES1 and DES2 are supposed to be equivalent to the iongels IL1 and IL2, respectively, due to their approximate ionic compositions and (2) the only difference between DES1–2 and DES4 is the HBD type. Note also that the conductivity of IL1 and IL2 is lower than DES equivalents, and the ionic conductivities of eutectogels are very similar (Figure S12); hence, performance differences cannot be explained by the ionic conductivity differences among electrolytes. We hypothesize that the strongly hydrogen-bonded Ch^+^ cations, which effectively dope the n-type polymer thin film in IL1 and IL2, may interact with the other component (lactic acid for DES1 and glycolic acid for DES2) and become too large, so they fail to dope the polymer and thus gate the channel effectively. It is also possible that the n-type film morphology is affected by the sugar alcohols in DES3 and DES4 (which is absent in DES1 and DES2), allowing the film to be doped by the eutectogel components. We note the increased OFF currents of this film in these electrolytes, as demonstrated in Figure S14, which may result from an improved intrinsic conductivity and pathways for ion transport.

### 2.4 Impact of Gel Type on the Quality of Electrophysiology Recordings

Having determined DES4 as the best-performing electrolyte, we next used the DES4-gated OECTs for the chronic acquisition of ECG signals. The switching speed of these devices is sufficient to capture electrophysiological signals in the frequency range from a few to hundreds of Hz, including ECG signals.^46,51,52^ We used an ECG simulator to generate the signals, which ensured the same signal quality throughout the measurements. The simulated ECG signals are always in the ideal state, thus free of any artifacts arising from acquisition conditions, the properties of human skin, and body movements. Having a consistent source signal is crucial when comparing the performance of different devices and allows us to evaluate device functionality during chronic recordings. To choose the channel material for this application, we first evaluated the shelf life and long-term operational stability of each channel gated with DES4. Figure S15 shows that while PEDOT:PSS channels lost current output over time, the enhancement mode devices operated with DES4 were very stable. Among the two types of stable channel materials, we chose p(g_3_C_2_T2-T) due to the higher transconductance values compared to the n-type device.

**Figure 7:**
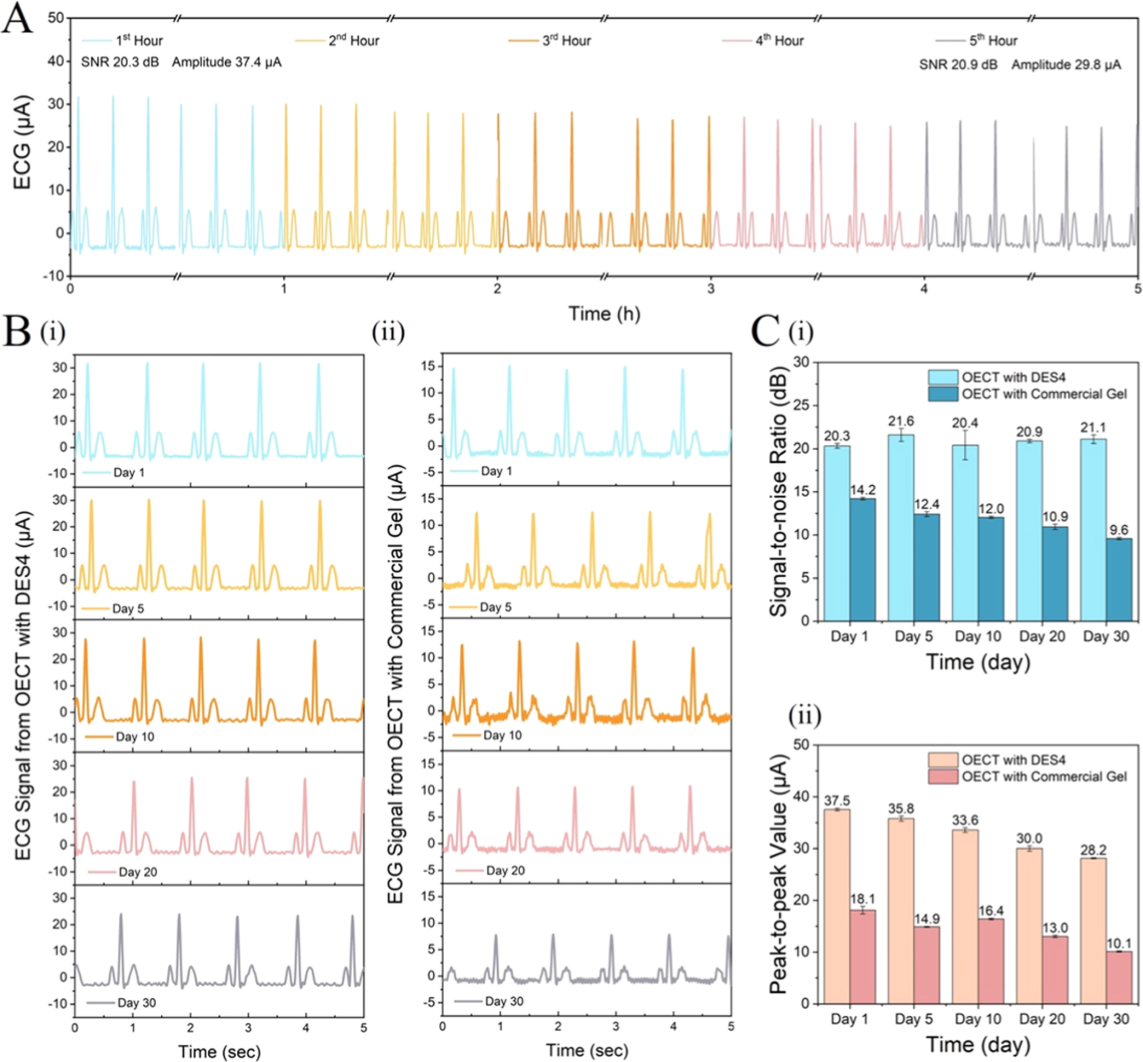
ECG acquisition with a DES4 eutectogel-gated p(g_3_C_2_T2-T) OECT. (A) Continuous ECG recordings for 5 h. (B) ECG signals acquired for 30 days using the same device gated with (i) DES4 and (ii) commercial gel. (C) ECG: (i) SNR and (ii) amplitude comparison.

We connected the ECG simulator along with a bias voltage (*V*_bias_), in series and between the gate and source of the OECT (Figure S16A). *V*_bias_ and *V*_D_ were set at −0.5 V to operate the p(g_3_C_2_T2-T) OECT, and the ECG signal was recorded as the *I*_D_. We designed two experiments to evaluate the performance of the DES4-gated p(g_3_C_2_T2-T) OECT in acquiring ECG signals. In the first case, we recorded ECG signals continuously for 5 h (Figure 7A) and calculated the signal-to-noise ratio (SNR) and the ECG peak amplitude (in current) to evaluate the signal quality. Figure S16 shows the ECG waveforms and frequency spectral output of the simulator and the same data recorded by the OECT. The OECT allowed for a high signal quality both at the beginning and at the end of the monitoring, with a 20.3% drop in amplitude (from 37.4 to 29.8 μA) yet no decrease in SNR (20.3 dB versus 20.9 dB).

In the second type of evaluation, we acquired ECG signals using the same OECT operated for 1 min per day for 30 days. Figure 7B-i shows the waveform sections for five of these days (days 1, 5, 10, 20, 30). To show the potential of DES4, we performed the same type of measurements using the same OECT but with the hydrogel extracted from a commercially available ECG electrode (Figure 7B-ii). The commercial gel-based ECG waveforms seem to have a higher level of noise and more amplitude attenuation compared to the signals from the DES4-gated OECT, especially after day 5. Figure 7C shows the SNR and amplitude variation of the two sets of signals, showing both higher SNR and amplitude of signals recorded by the DES4-gated OECTs. Over the 30 days, the SNR of the DES4-gated OECT remained constant (*p* < 0.01), while the SNR of the commercial gel-gated device decreased by 33% (*p* < 0.001, Figure S16D-i). We note a 25 and 44% decrease in the ECG amplitude for the DES4- and commercial gel-gated OECTs, respectively (*p* < 0.001, Figure S16D-ii). The better performance of the eutectogel-integrated device is attributed to the high mechanical durability and nonvolatile nature of the gel, which makes it more stable for prolonged use. When subjected to 100 consecutive output characteristic recordings, the device with the commercial gel showed an *I*_D_ drop of 17.3% (Figure S16E-i), while the DES4-gated device remained stable. The current generated by the same channel with the DES4 gating is also higher, suggesting a correlation between the ECG signal quality and ON current values.

## 3 Conclusions

In this work, we tested the performance of seven nonliquid electrolytes with distinct ionic components (PBS, biocompatible ionic liquids, and biocompatible DES) as the dielectric medium for OECTs. The results unequivocally establish that the eutectogel electrolyte, made of poly(DGLY) as the polymer matrix and choline chloride (ChCl) and 1,3-propanediol deep eutectic solvent as the ionic component, emerges as the superior choice for Ag/AgCl-gated OECTs. This holds true across a spectrum of the OECT, encompassing p-type depletion and p-type and n-type enhancement mode devices. Through electrochemical characterizations, we elucidated that the superiority of the eutectogel as an OECT electrolyte stems from its effects on the channel, as observed in the electrochemical potentials, oxidation/reduction potentials in CV curves, and electrochemical impedance profiles. If the electrolyte ions do not have any barrier to entering the polymer film, most device characteristics are governed by the electrochemical potential of the gate electrode in that particular electrolyte, which mainly affects the threshold voltages. We showed that the best-performing OECT, the eutectogel-gated p-type enhancement mode device, exhibits exceptional stability in long-term ECG signal monitoring, with no decrease in SNR even after a continuous 5 h monitoring session or over a span of 30 days with daily measurements. Furthermore, this eutectogel-gated device surpassed devices utilizing commercial hydrogels as electrolytes in terms of signal amplitude and SNR. We unveil the immense potential of eutectogels as a nonaqueous gel-based electrolyte for acquiring physiological signals, with particular promise for applications demanding highly stable and long-term monitoring capabilities.

## 4 Experimental Section

### 4.1 Materials

The materials used for gel synthesis are choline bicarbonate (80% in H_2_O), choline chloride (ChCl, >98%), lactic acid, glycolic acid, glycerol, 1,3-propanediol, 2-hydroxyethyl methacrylate (HEMA, 97%), ethylene glycol dimethacrylate (EGDMA, 98%), glycerol 1,3-diglycerolate diacrylate (DGLY), and 2-hydroxy-2-methylpropiophenone (97%), all of them purchased from Sigma-Aldrich. PEDOT:PSS (PH1000) was received from Heraeus. p(C_6_NDI-T) and p(g_3_C_2_T2-T) were synthesized using existing protocols.^40,53^ 1× PBS solution was prepared following the manufacturer’s instructions (Merck). It contained about 137 × 10^–3^ M of NaCl, 2.7 × 10^–3^ M of KCl, 10 × 10^–3^ M of Na_2_HPO_4_, and 1.8 × 10^–3^ M of KH_2_PO_4_. The Ag/AgCl electrode used as the gate was purchased from Warner Instruments, LLC, Holliston, MA. The 1-ethyl-3-methylimidazolium bis(trifluoromethylsulfonyl)imide ([EMIM][TFSI]) ionic liquid and poly(vinylidene fluoride-*co*-hexafluoropropene) (PVDF-HFP) were purchased from Sigma-Aldrich. All aqueous solutions were prepared with ultrapure water (Milli-Q, Millipore). All chemicals were used without further purification.

### 4.2 Ionic Component Synthesis

The cholinium-based ILs used in this work, namely, cholinium lactate ([Ch][Lac]) and cholinium glycolate ([Ch][Glyco]), were prepared by the dropwise addition of the corresponding acid (1:1 molar ratio) to the aqueous choline bicarbonate. The mixtures were stirred at room temperature for 12 h. The resulting products were washed three times with diethyl ether to remove the unreacted acid. The excess water and traces of other volatile substances were removed by rotary evaporation and a dry vacuum. The cholinium-based DESs (ChCl:lactic acid, ChCl:glycolic acid, ChCl:glycerol, and ChCl:1,3-propanediol) were synthesized by mixing choline chloride with the corresponding acid (1:2 molar ratio) and heating up to 90 °C with magnetic stirring until a homogeneous liquid was formed. The pH values of the ionic components of IL1 and IL2 were measured using pH strips (Whatman WHA10362000 indicator papers). The pH values of PBS and the ionic components of HG, DES1, DES2, DES3, and DES4 were measured using a 781 pH/Ion Meter (Metrohm AG, Herisau, Switzerland).

### 4.3 Gel Synthesis

The hydrogel was synthesized, adding the corresponding saline aqueous solution (0.5 M PBS, equivalent to 3.5× PBS, 50 wt %) to HEMA:EGDMA (95:5 mass ratio, 50 wt %). 2-Hydroxy-2-methylpropiophenone photoinitiator was added in 0.1 wt % to this mixture, and the mixture was photopolymerized with UV light in a silicon mold (a diameter of 8 mm and a height of 0.5 mm). For iongel and eutectogel preparation, ILs and DESs (30 wt %) were mixed with DGLY (70 wt %) and photopolymerized in the same way as hydrogels. All samples were stored in the refrigerator to avoid water evaporation. For the [EMIM][TFSI]/PVDF-HFP gel, [EMIM][TFSI] ionic liquid and PVDF-HFP (4:1 w/w) were dissolved in acetone with the following proportions: 17.6 wt % ionic liquid, 4.4 wt % PVDF-HFP, and 78 wt % acetone. PVDF-HFP was initially dissolved in acetone at 60 °C for 10 h with continuous stirring. Once it completely dissolved, [EMIM][TFSI] was introduced into the solution and allowed to mix for an additional 2 h.

### 4.4 Device Fabrication

The OECTs were microfabricated on the 4 in. glass wafers based on established protocols using standard photolithography and parylene C (PaC) peel-off techniques. A first layer of photoresist (AZ5214) was spin-coated and exposed to ultraviolet light using a contact aligner. The photoresist patterns were generated with an AZ 726 developer, followed by metal sputtering of 10 nm Cr and 100 nm Au and a standard lift-off process using hot dimethyl sulfoxide to create electrodes and interconnection pads. A second layer of photoresist AZ9260 was coated on the substrates and later developed using an AZ developer. A parylene C layer was deposited to insulate the Au interconnects. The OECT channel was patterned by reactive ion etching using a second layer of parylene C that was peeled off to yield the patterns. The aqueous dispersion of PEDOT:PSS containing ethylene glycol (5 vol %), sodium dodecylbenzenesulfonate (0.25 vol %), and (3-glycidyloxypropyl)trimethoxysilane (1 wt %) was sonicated for 30 min and then spin-coated (3000 rpm; 45 s) on the substrates, leading to a film thickness of ca. 160 nm. The PEDOT:PSS OECTs were annealed at 140 °C for 1 h to activate GOPS and avoid dissolution of the polymer film in aqueous medium. The p(g_3_C_2_T2-T) and p(C_6_NDI-T) films were spin-coated (800 rpm; 45 s) from a chloroform solution (5 g/L) on the substrates to yield a film thickness of ca. 85 nm in the channel. All devices were rinsed with deionized water before use.

### 4.5 Device and Film Characterization

All measurements were conducted under ambient atmosphere conditions. The steady-state characteristics of the transistors were recorded by using a Keithley 2602A type source meter unit operated by a customized LabVIEW software. The drain (*V*_D_) and gate (*V*_G_) voltages were applied while the source electrode was the common ground. All gel samples used as the OECT electrolytes were prepared as cylinders with a diameter of 8 mm and a thickness of 0.5 mm. The 1× PBS was used as a liquid electrolyte OECT with a volume of 50 μL. The channel (*I*_D_) and gate currents (*I*_G_) were simultaneously monitored. The OECT threshold voltage (*V*_TH_, pinch-off voltage for PEDOT:PSS, *V*_P_) was extracted from *I*_D_^1/2^ vs *V*_G_ plots. The operational stability test of PEDOT:PSS-based OECTs was conducted at a *V*_D_ of −0.5 V. *V*_G_ was switched between 0.2 and −0.4 V for 10 s each over 1 h of electrochemical cycling. For the p(g_3_C_2_T2-T)-based OECT, the same protocol applied but *V*_D_ was at −0.5 V and *V*_G_ was switched between 0 and −0.5 V. For the p(C_6_NDI-T)-based OECT, *V*_D_ was 0.5 V and *V*_G_ was switched between 0 and 0.5 V. The OECT response time (τ) was extracted by fitting the rising curve of *I*_D_ versus time with an optimized exponential fit *y* = *a*·(1 – exp(−*bx*))·exp(*cx*), where *y* is the channel current, *x* is the time, *a*, *b*, *c* are the three fitting parameters, and τ = 1/*b*.^46^

We assigned an OECT channel to evaluate the performance of each combination of the gel electrolyte and channel material, and the statistics for each combination were generated by conducting multiple measurements on this single device. To exclude any effects from the device-to-device differences, we first measured the output characteristics of each OECT channel in PBS before characterizing the device with the gel as the electrolyte. Only those devices with deviations in ON currents within 5% were further selected for gel electrolyte measurements.

Electrochemical measurements were conducted with a Biologic VSP-3e Potentiostat. A square Au electrode with dimensions of 3 × 3 mm^2^ coated with the polymer films was used as the working electrode. The leakless Ag/AgCl electrode and the Pt wire were used as the reference and counter electrodes, respectively. Another circular Au electrode (diameter = 4 mm) was used to estimate the conductivities of PBS and the gels based on the EIS measurements, with the relation of , where σ is the gel conductivity, *R*_gel_ is the gel resistance from EIS, and ϕ is the diameter of the Au electrode.^54^ During EIS measurements, we set the operational potential of the EIS to the equivalent of the OECT *V*_G_ corresponding to the maximum *g*_m_ for each film/electrolyte couple.

### 4.6 ECG Measurements

One channel of lead-II ECG (among the 12-lead standard ECG) signal was generated using the AECG100 ECG simulator (WHALETEQ Co., LTD) with a 2 mV amplitude and a 60 bpm input. The simulator along with a bias voltage (*V*_bias_) was connected in series and between the gate (Ag/AgCl) and the source of the OECT. *V*_bias_ and *V*_D_ were set at −0.5 V by the Keithley 2602A source meter unit. We acquired the ECG signal from the *I*_D_ of the OECT. In 1 min per day for a 30 day experiment, the signal was acquired and restored by customized LabVIEW software. In the 5 h continuous acquisition experiment, the signal was acquired using an RHD2000 Evaluation System (Intan Technologies, LA) and restored simultaneously and directly into the computer disk through its RHD USB Interface GUI Software. The commercial gel was detached from the ECG monitoring electrodes (Graphic Controls Data Recording, Buffalo, NY). We removed the DC component of the *I*_D_ to obtain the 0-mean ECG signal and performed no further digital signal processing on the data. The SNR was calculated from the ratio of the peak-to-peak current amplitude of the ECG signal to that of the noise region.

## Supporting Information Available

pH values of PBS and all gel samples, the (semi)conducting polymers used in the OECT channel, photographs of all gel samples, the output characteristics of hydrogel-gated and PBS-gated OECTs, comparison of OECT response time of the hydrogel-gated device versus the PBS-gated one, the output characteristics of iongel-gated OECTs, the output characteristics of p(C_6_NDI-T) OECTs gated with [EMIM][TFSI] ionic liquid and [EMIM][TFSI]/PVDF-HFP ionic gel, OECT response time of all devices, the output characteristics of eutectogel-gated OECTs, comparison of OECT response time of the eutectogel-gated device versus the PBS-gated one, a summary of OECT metrics for various channels and electrolyte combinations, OECT performance comparison (PBS versus DES4 gating), the ionic conductivity of all electrolytes, the electrochemical potential changes of the OECT terminals, the transfer curves of the eutectogel or PBS-gated OECTs in log scale, the 1 month long OECT current stability with PBS or DES4 used as the electrolyte, and ECG acquisition setup and analysis (PDF)
