# Supplementary material for: Eutectogels as a Semisolid Electrolyte for Organic Electrochemical Transistors
Source: Chem Mater. 2024 Feb 5;36(4):1841–54. doi: 10.1021/acs.chemmater.3c02385 (PMC10902863; doi:10.1021/acs.chemmater.3c02385)
Supplement: Supplementary file 1 — cm3c02385_si_001.pdf [file cm3c02385_si_001.pdf]

Supporting Information for

Eutectogels as a Semi-Solid Electrolyte for Organic Electrochemical Transistors

Yizhou Zhong[1], Naroa Lopez-Larrea[2], Marta Alvarez-Tirado[2], Nerea Casado[2,3], Anil Koklu[1], Adam Marks[4], Maximilian Moser[4], Iain McCulloch[4], David Mecerreyes[2,3], Sahika Inal[1*]

[1] *Organic Bioelectronics Laboratory, Biological and Environmental Science and Engineering Division, King Abdullah University of Science and Technology (KAUST), Thuwal 23955-6900, Saudi Arabia.*

[2] *POLYMAT, University of the Basque Country UPV/EHU, Avenida Tolosa 72, Donostia-San Sebastian, Guipuzcoa 20018, Spain.*

[3] *IKERBASQUE, Basque Foundation for Science, Plaza Euskadi 5, Bilbao 48009, Spain.*

[4] *Department of Chemistry, University of Oxford, Oxford OX1 3TF, UK.*

Corresponding Author: sahika.inal@kaust.edu.sa

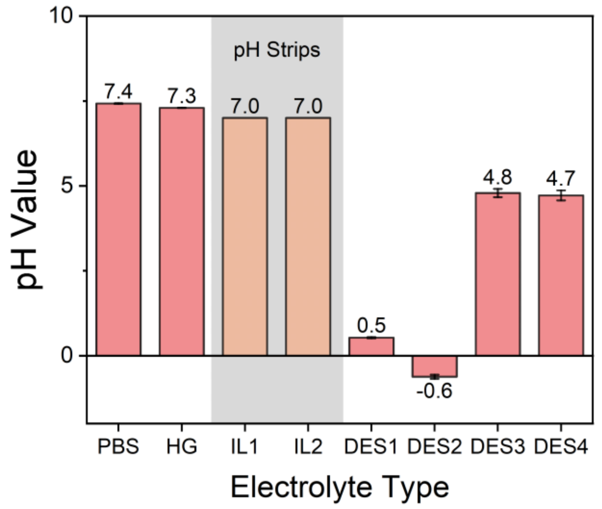

Figure S1. pH values of PBS and all gel samples (before crosslinking). The ionic liquids were tested using pH strips, while a pH meter was used for the other electrolytes.

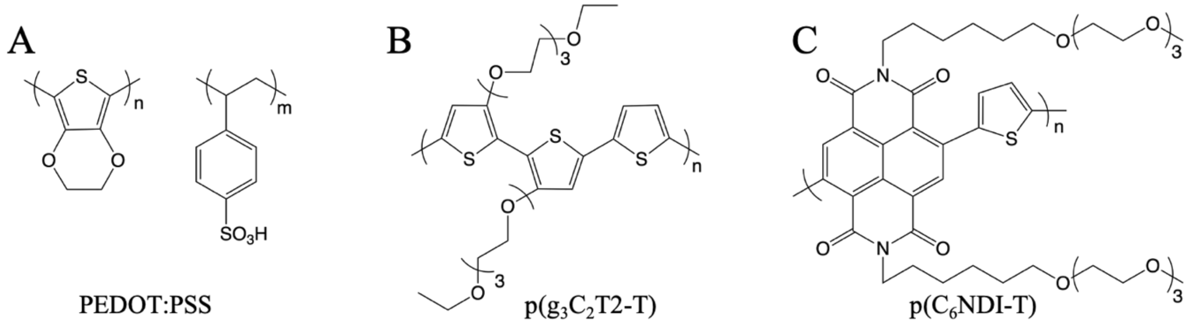

Figure S2. The (semi)conducting polymers used in the OECT channel: **A)** PEDOT:PSS, **B)** p(g_3C_2T2-T), and **C)** p(C_6NDI-T).

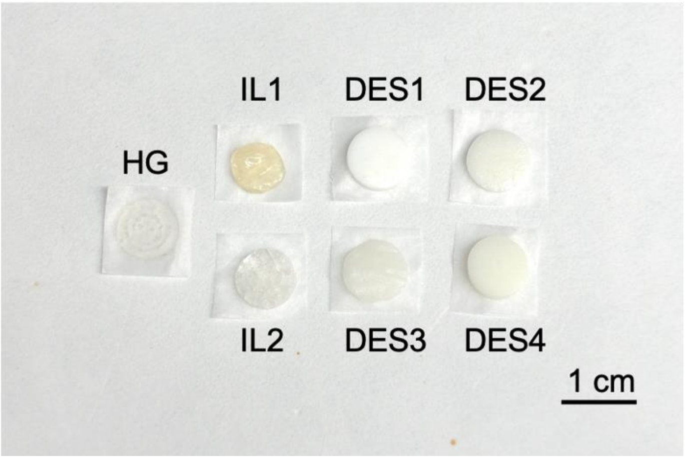

Figure S3. Photographs of all gel samples used in this work.

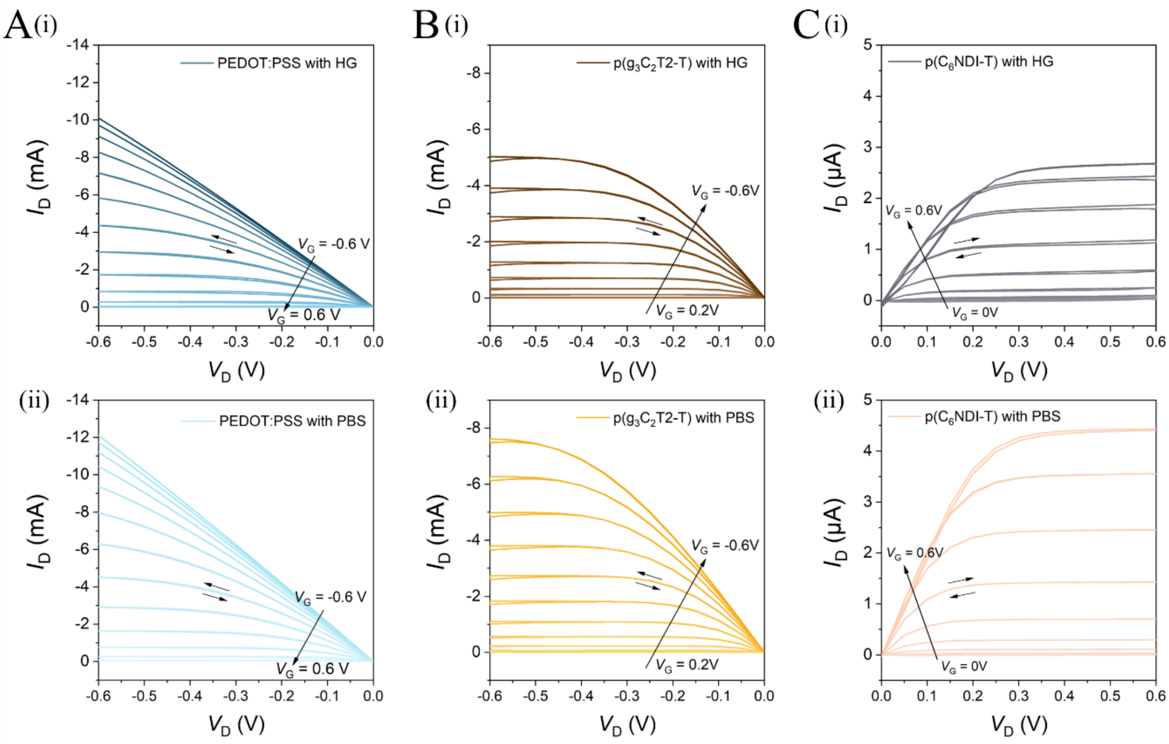

Figure S4. The output characteristics of hydrogel-gated OECTs (top) and PBS-gated OECTs (bottom) with channels made of **A)** PEDOT:PSS, **B)** p(g$_3$C$_2$T2-T), and **C)** p(C$_6$NDI-T).

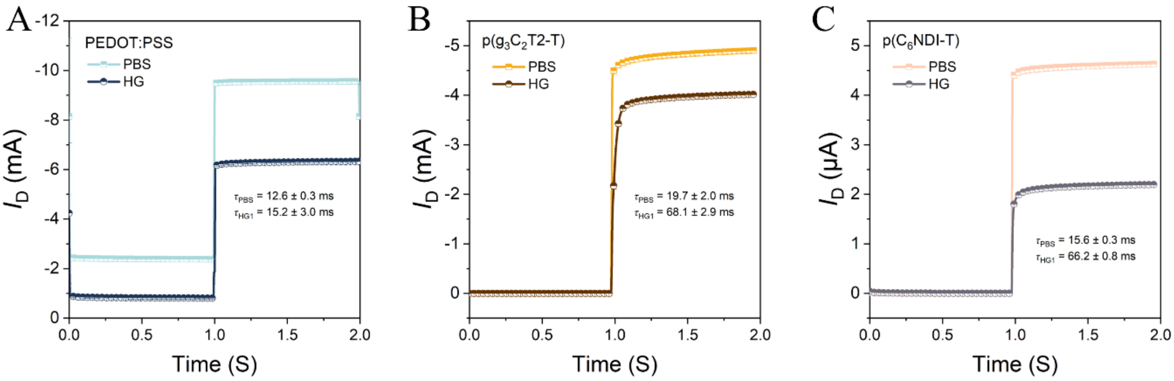

Figure S5. Comparison of OECT characteristics of the hydrogel-gated device versus the PBS-gated one. The real-time change in I_D as a response to V_G pulse, showing the response time (τ) in each electrolyte condition, with channels made of **A)** PEDOT:PSS, **B)** p(g$_3$C$_2$T2-T), and **C)** p(C$_6$NDI-T). V_D = -0.5 V for p-type OECTs and V_D = 0.5 V for n-type devices.

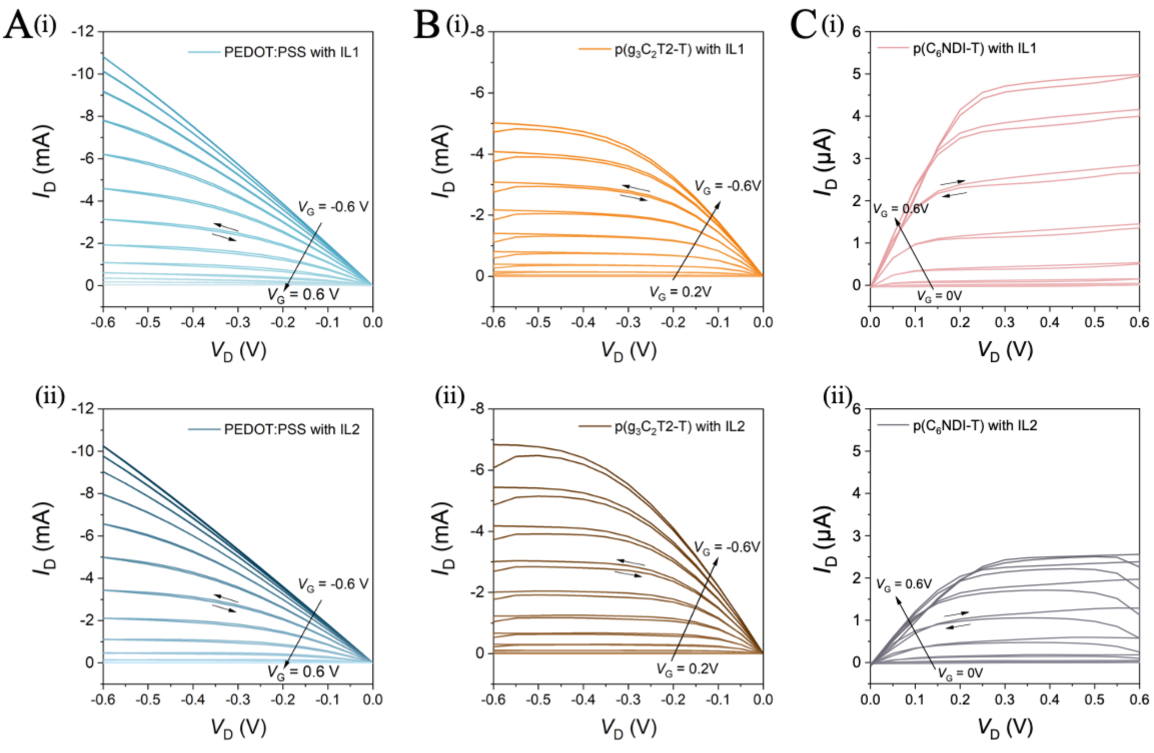

Figure S6. The output characteristics of iongel-gated OECTs using IL1 (top) IL2 (bottom) as the solid-state electrolyte. The channel material is **A)** PEDOT:PSS, **B)** p(g_3C_2T2-T), or **C)** p(C_6NDI-T).

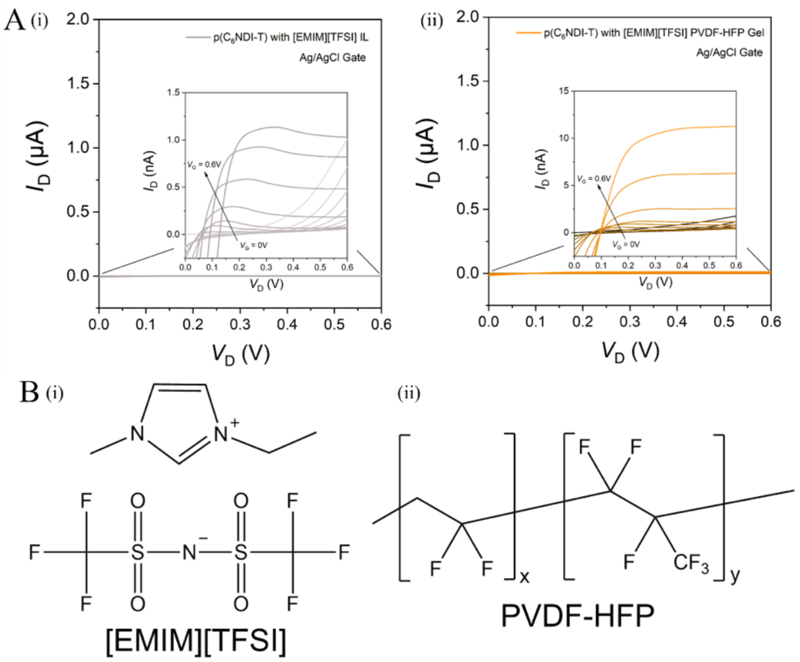

Figure S7. A) The output characteristics of p(C_6NDI-T) OECTs gated with (i) 1-ethyl-3-methylimidazolium bis(trifluoromethylsulfonyl)imide ([EMIM][TFSI]) ionic liquid (IL) and (ii) [EMIM][TFSI]/poly(vinylidene fluoride-co-hexafluoropropene) (PVDF-HFP) ionic gel, with Ag/AgCl as the gate. **B)** The chemical structures of (i) [EMIM][TFSI] and (ii) PVDF-HFP.

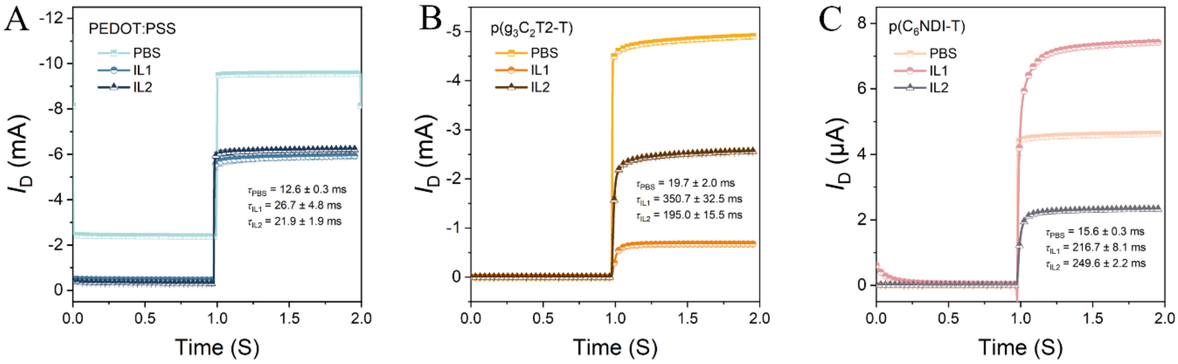

Figure S8. The real-time change in iongel-gated OECT current and the one gated with PBS in response to a V_G pulse. The calculated τ are given for each gate electrolyte condition, with OECT channels made of **A)** PEDOT:PSS, **B)** p(g_3C_2T2-T), and **C)** p(C_6NDI-T). All characteristics were recorded at V_D = -0.5 V for p-type OECTs and V_D = 0.5 V for n-type devices.

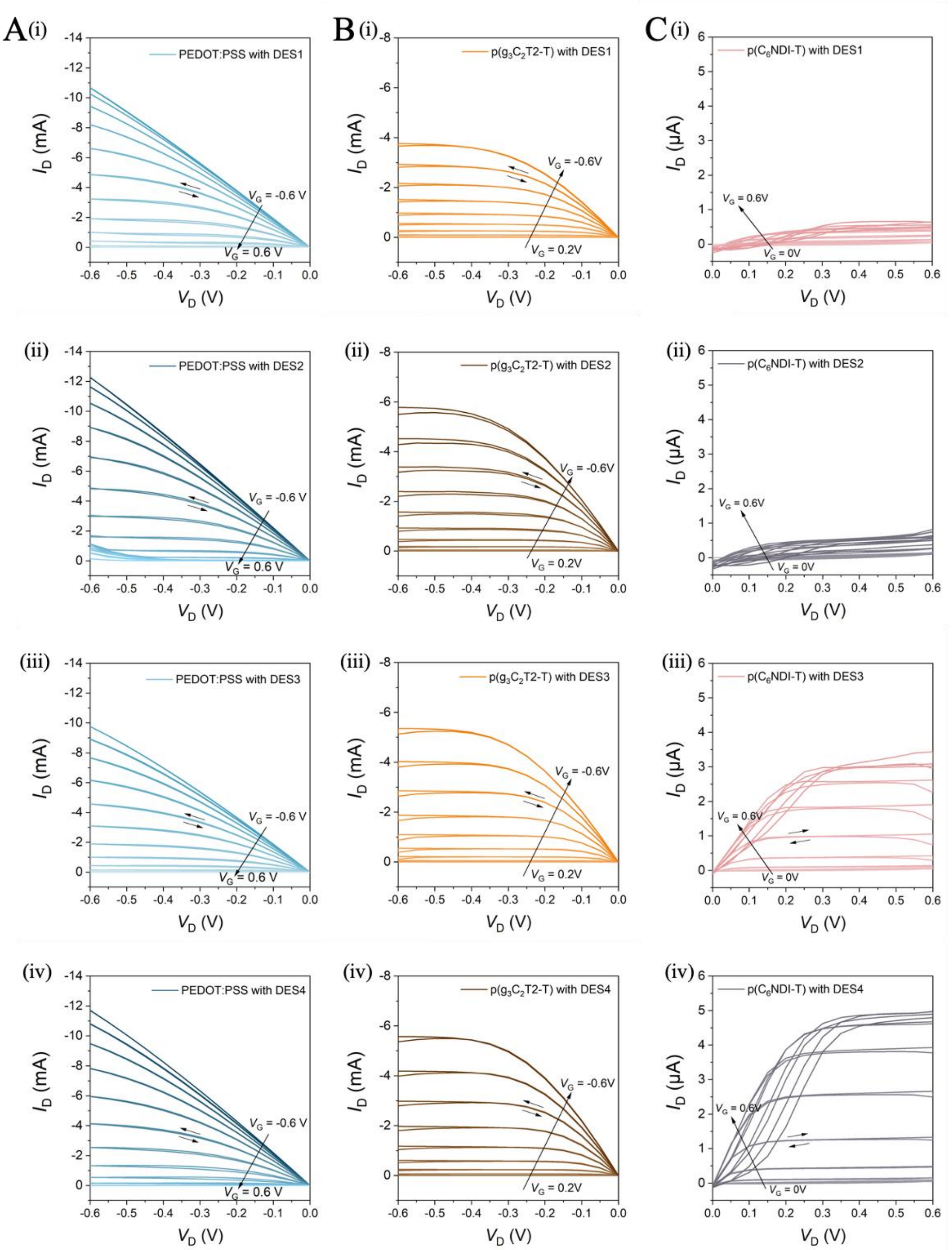

Figure S9. The output characteristics of eutectogel-gated OECTs **A)** PEDOT:PSS with (i) DES1, (ii) DES2, (iii) DES3, and (iv) DES4, **B)** p(g_3C_2T2-T) with (i) DES1, (ii) DES2, (iii) DES3, and (iv) DES4, and **C)** p(C_6NDI-T) with (i) DES1, (ii) DES2, (iii) DES3, and (iv) DES4.

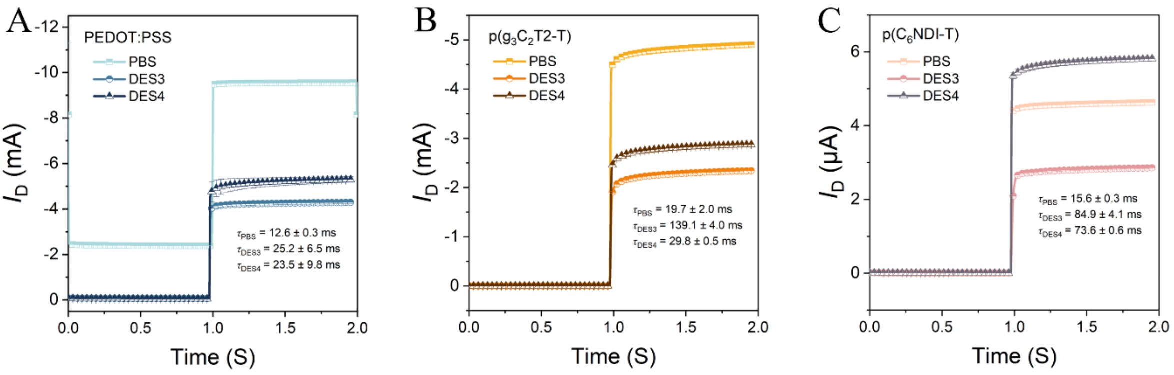

Figure S10. The transient current response of eutectogel or PBS gated channels to a V_G pulse. The switching speeds for each electrolyte type are shown for the channel made of **A)** PEDOT:PSS, **B)** p(g_3C_2T2-T), and **C)** p(C_6NDI-T). All characteristics were recorded at V_D = -0.5 V for p-type OECTs and V_D = 0.5 V for n-type devices.

Table S1. A summary of OECT metrics for various channel and electrolyte combinations

Gel	Max. I_D (mA)			Max. g_m (mS)		
	PEDOT:PSS	p(g_3C_2T2-T)	p(C_6NDI-T)	PEDOT:PSS	p(g_3C_2T2-T)	p(C_6NDI-T)
PBS	-9.95 ± 0.15	-7.38 ± 0.04	(5.41 ± 0.62) × 10^{-3}	14.9 ± 0.2	24.8 ± 0.1	(25.7 ± 2.3) × 10^{-3}
HG	-7.93 ± 0.23	-5.10 ± 0.05	(2.86 ± 0.12) × 10^{-3}	12.0 ± 0.1	23.5 ± 0.1	(14.2 ± 0.2) × 10^{-3}
IL2	-7.99 ± 0.35	-5.74 ± 0.58	(2.57 ± 0.05) × 10^{-3}	13.2 ± 0.2	25.0 ± 0.9	(13.6 ± 0.2) × 10^{-3}
DES4	-9.22 ± 0.26	-5.42 ± 0.05	(5.55 ± 0.38) × 10^{-3}	15.5 ± 0.8	27.3 ± 0.2	(29.9 ± 1.9) × 10^{-3}
	**ON/OFF Ratio**			**τ (ms)**		
	**PEDOT:PSS**	**p(g_3C_2T2-T)**	**p(C_6NDI-T)**	**PEDOT:PSS**	**p(g_3C_2T2-T)**	**p(C_6NDI-T)**
PBS	(2.4 ± 0.1) × 10^2	(3.1 ± 0.7) × 10^4	(1.7 ± 0.7) × 10^3	12.6 ± 0.3	19.7 ± 2.0	15.6 ± 0.3
HG	(1.4 ± 0.1) × 10^4	(2.4 ± 0.4) × 10^4	(6.4 ± 1.0) × 10^1	15.2 ± 3.0	68.1 ± 2.9	66.2 ± 0.8
IL2	(2.1 ± 0.4) × 10^4	(3.3 ± 0.5) × 10^4	(2.7 ± 0.5) × 10^2	21.9 ± 1.9	195.0 ± 15.5	249.6 ± 2.2
DES4	(3.5 ± 0.1) × 10^5	(2.4 ± 1.5) × 10^5	(1.6 ± 0.7) × 10^2	23.5 ± 9.8	29.8 ± 0.5	73.6 ± 0.6
	**I_D Retention after 1 hour (%)**			**V_{TH} or V_P (V)**		
	**PEDOT:PSS**	**p(g_3C_2T2-T)**	**p(C_6NDI-T)**	**PEDOT:PSS**	**p(g_3C_2T2-T)**	**p(C_6NDI-T)**
PBS	93.9	85.2	95.7	0.61	-0.13	0.26
HG	73.4	90.0	99.4	0.51	-0.18	0.26
IL2	100.6	65.4	103.1	0.42	-0.20	0.25
DES4	94.7	101.4	103.1	0.33	-0.23	0.22

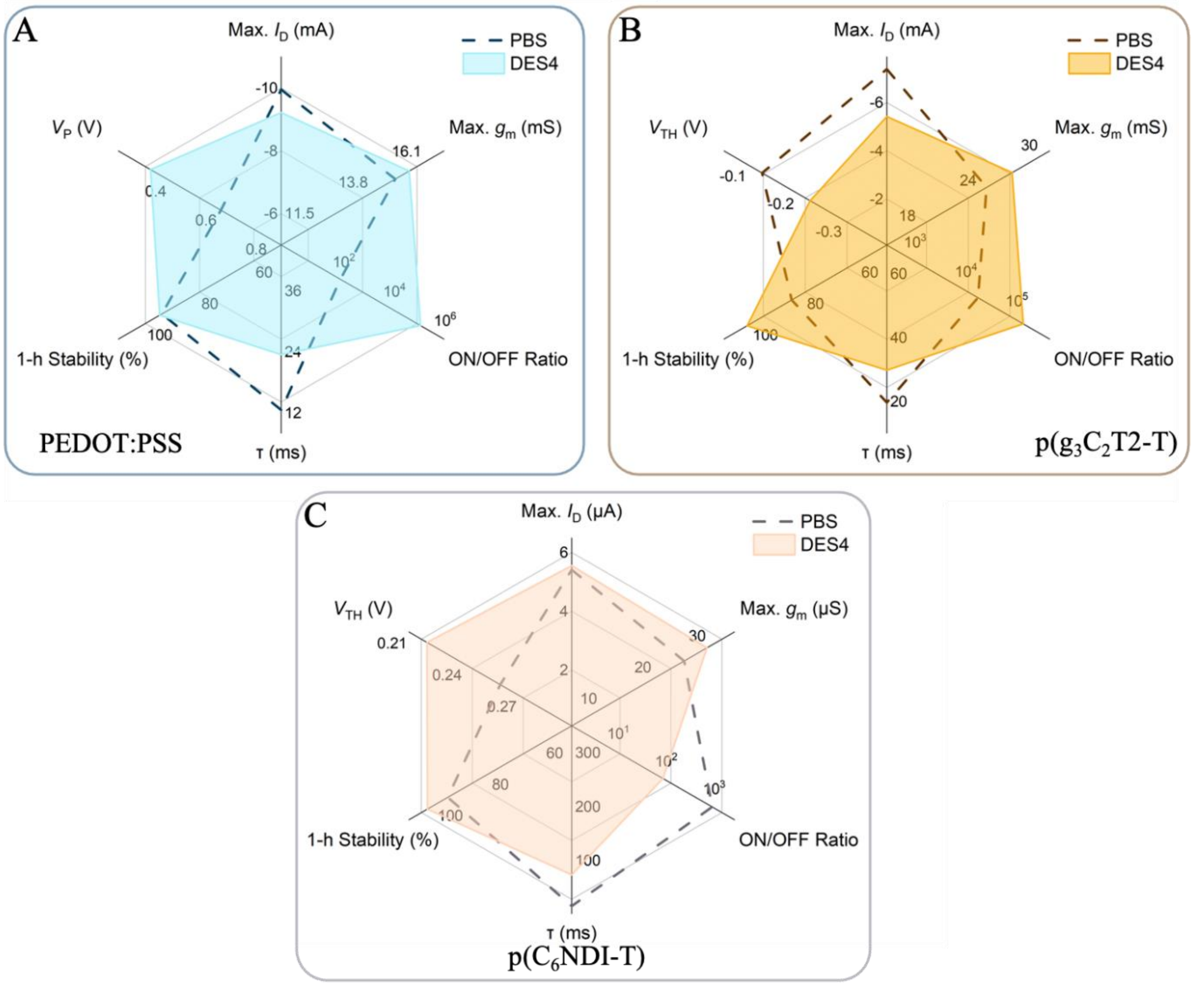

Figure S11. The performance metrics comparison of OECTs gated with PBS and DES4. The channel is **A)** PEDOT:PSS, **B)** p(g_3C_2T2-T), or **C)** p(C_6NDI-T).

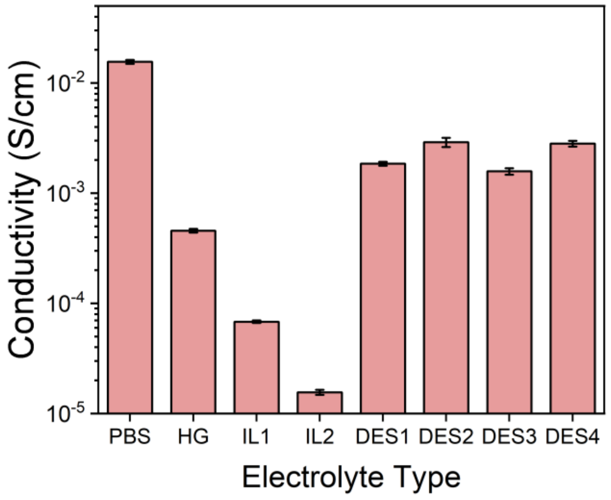

Figure S12. The ionic conductivity of PBS and all gel samples.

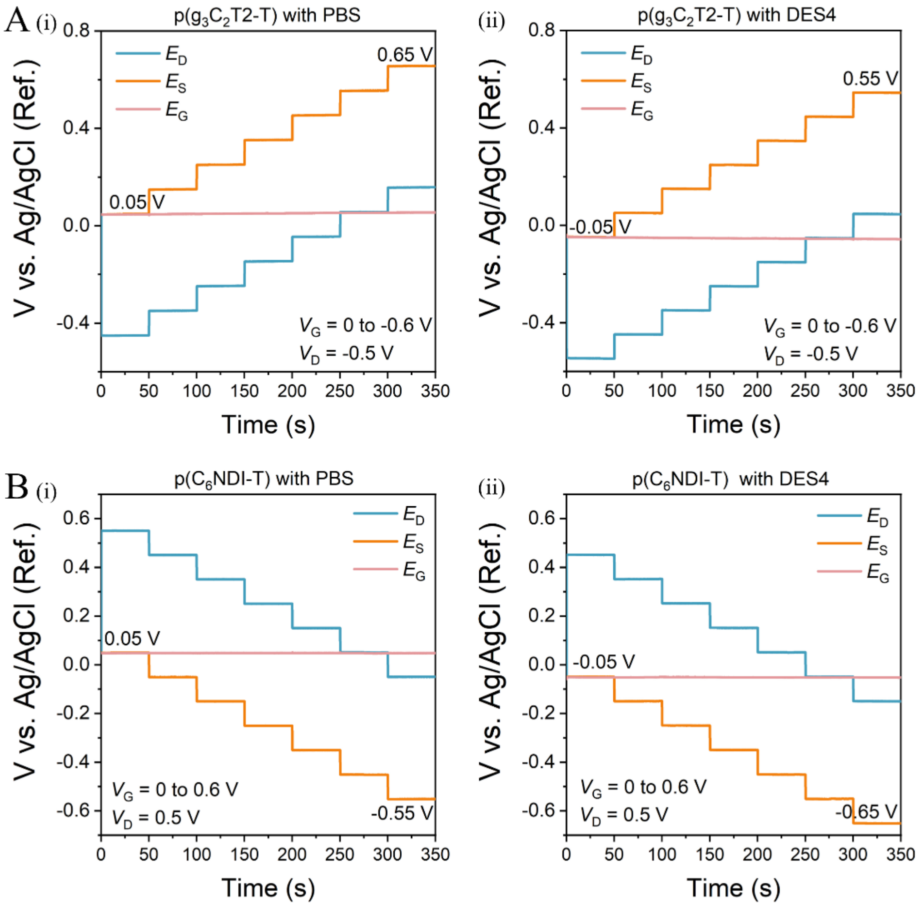

Figure S13. The electrochemical potential changes of the terminals of **A)** p(g_3C_2T2-T) and **B)** p(C_6NDI-T) OECTs measured during device operation in (i) PBS and (ii) DES4 eutectogel. V_D was at 0.5 V, and V_G changed from 0 to 0.6 V with a step of 0.1 V. The gate potential at the beginning of the measurement (E_G) and the source potential at V_G = 0.6 V (E_S) are labeled.

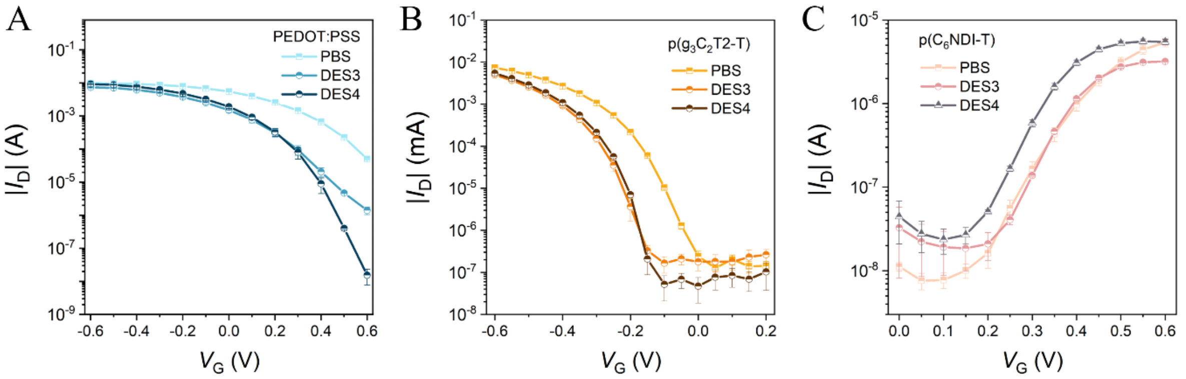

Figure S14. The transfer curves of the eutectogel-gated OECTs (and the PBS-gated ones) in log scale. The channel is **A)** PEDOT:PSS, **B)** p(g_3C_2T2-T), and **C)** p(C_6NDI-T). All characteristics were recorded at V_D = -0.5 V for p-type OECTs and V_D = 0.5 V for n-type devices.

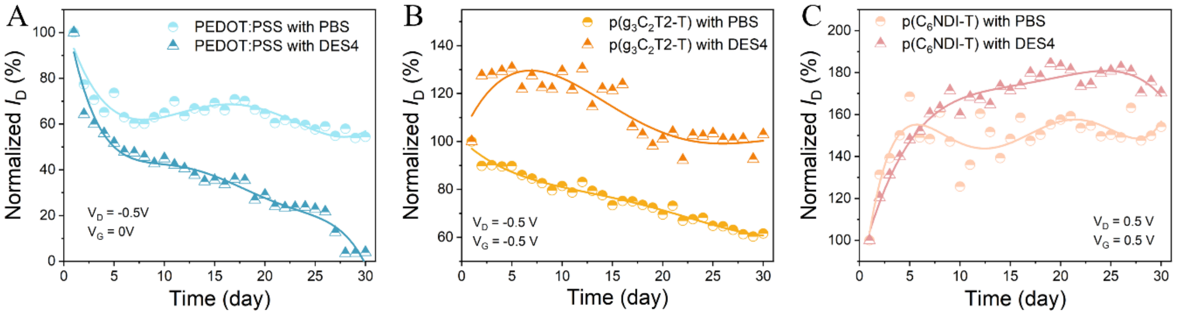

Figure S15. The one month-long OECT current stability (I_D values are normalized by the current measured on the first day) with PBS or DES4 used as the electrolyte for **A)** PEDOT:PSS, **B)** p(g_3C_2T2-T), and **C)** p(C_6NDI-T) channel.

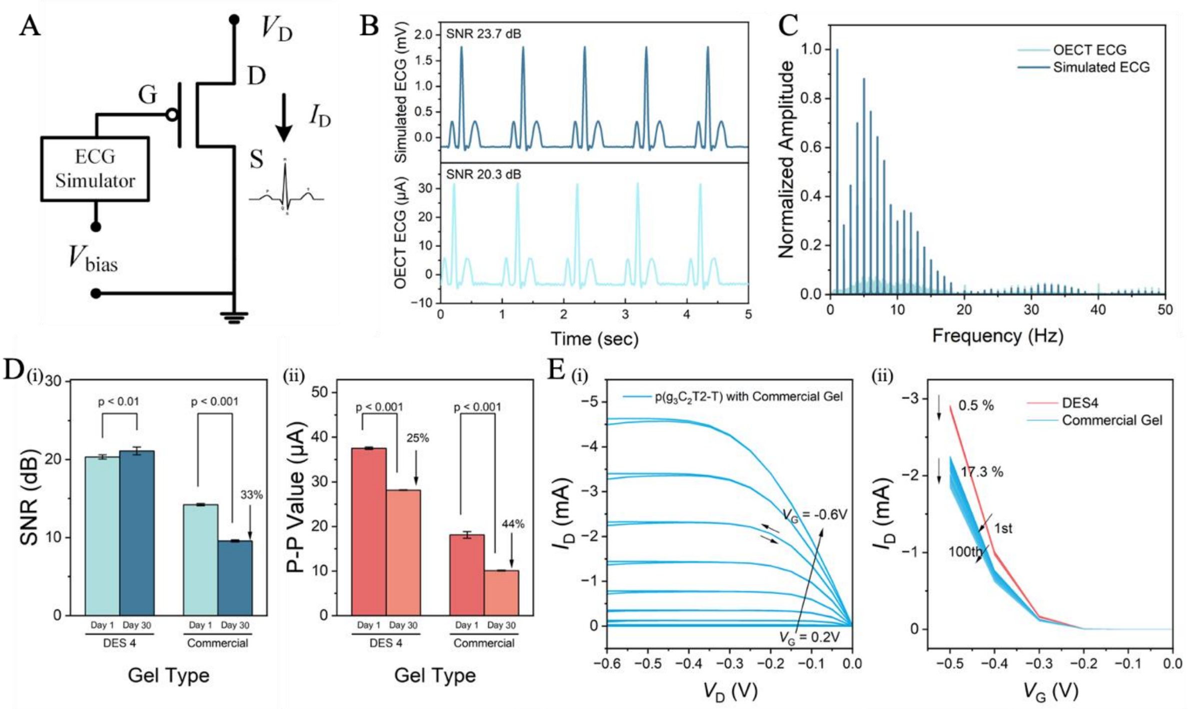

Figure S16. ECG acquisition setup and analysis. A) The circuit diagram for the ECG acquisition using the OECT. **B)** The ECG signals from the simulator and the same signals acquired using the p(g_3C_2T2-T) OECT operated with DES4 electrolyte ($V_D = V_{bias}$ = -0.5 V). **C)** The normalized frequency spectra of both simulated and OECT ECG processed using the fast Fourier transform function. The spectra were normalized using the peak value. **D)** Paired comparison of (i) signal-to-noise ratio, and (ii) peak-to-peak current amplitude of p(g_3C_2T2-T) channel gated by the commercial gel and DES4. **E)** The output characteristics of the p(g_3C_2T2-T) OECT gated by the commercial gel (i) and the 100 consecutive transfer curves (V_D = -0.5 V) of the same channel gated with DES4 or the commercial gel (ii)
